# Wrack line formation and composition on shores of a large Alpine lake: The role of littoral topography and wave exposure

**DOI:** 10.1371/journal.pone.0294752

**Published:** 2023-11-30

**Authors:** Wolfgang Ostendorp, Hilmar Hofmann, Jens Peter Armbruster

**Affiliations:** 1 Environmental Physics Group, Limnological Institute, University of Konstanz, Konstanz, Germany; 2 Staff Unit Sustainability, University of Konstanz, Konstanz, Germany; 3 Institute for Landscape Ecology and Nature Conservation (ILN) Südwest, Kirchheim u. T., Germany; King’s College London, UNITED KINGDOM

## Abstract

Wrack lines are a key formation along shorelines that provide organic matter and bring ecological diversity to the local environment. Although wrack line formation has been extensively studied along marine beaches and estuaries, in contrast, knowledge about the environmental variables that promote wrack line formation within inland lakes is widely lacking. In one of the first studies to focus on wrack line formation on lakesides, we analysed the dimensions, volume, elevation and particulate composition of 36 wrack lines across 20 shore sections of a large, oligotrophic Alpine lake with natural water level fluctuations (Lake Constance-Obersee). Using multivariate partial least squares (PLS) regression, we identified the key environmental variables that drive wrack accumulation in lakeside areas. Our results demonstrate that wrack line volume increased with (1) the width of the eulittoral zone as an indicator of the swash conditions (up-rush vs. down-wash), (2) high exposure to wind waves as indicated by the total effective fetch, (3) high exposure to ship waves (catamaran ferry), and (4) the width of the sublittoral zone as an indicator of the availability of source material (*Chara* spp.) and of the wave energy dissipation rate of the incoming deep water waves. Sediment texture played only a minor role. Wide eulittoral zones and high ship wave exposure favoured high proportions of lake-borne components (*Chara* remains, mollusc shells), while the reverse was true for land-based components. Anthropogenic wastes were only present in small proportions. We discuss four main factor groups influencing the amount of wrack in marine beaches and on lakeshores considering similarities (waves, breakers, swash, dissipation, relief) and differences (tides vs. annual water level fluctuations) of the two systems, and point out research gaps. We demonstrate that wrack line formation is also important in large inland lakes and can be analysed using basic ideas from relevant marine studies.

## Introduction

The shores of Central European lakes are ecologically important ecotones between terrestrial and pelagic habitats [1–7], the carbon budget of which is controlled by inputs from different sources.

The net primary production from sublittoral meadows of submerged macrophytes [8–12] and the eulittoral reeds [13] overlap with the carbon input from the riparian forests (foliage, branch wood) [14–17] and from the pelagic zone, such as phytoplankton blooms [18–21].

Carbon matter in this zone becomes mineralised, either in situ or below the wave base after fragmentation, or is transferred to the permanent sediment [13]. However, some material remains in the littoral zone, where it can temporally amass as wrack lines (overview: [6]).

Our current knowledge of wrack fringes has been prominently drawn from marine coasts and river banks, where they aggregate during strong onshore winds and high water levels. Wrack lines remain on shore even after wave action has died down and the water level has receded, and develop their own structural elements and habitat types (overviews: [6, 22–24]).

These marine wracks consist of varying amounts of torn plant material (macroalgae, seagrass, reed stems and salt marsh plants), animal remains (e.g. mollusc shells), driftwood washed in via tributaries, and anthropogenic waste, particularly plastics [25–34]. Wrack line formation is dependent on (1) the availability of suitable material and its physical properties (e. g. buoyancy), (2) hydrodynamic transport conditions, both offshore (waves, nearshore currents) and nearshore (surf and wave run-up) [35–37], and (3) shore morphology (inclination, hydraulic roughness) [31, 35, 38–40]. Seasonality of weather conditions can additionally drive fluctuations in wave and water level conditions, and the availability of source material [28, 32, 36].

In comparison, wrack line formation on shores of inland lakes has received by far less attention until now (e.g. [41–45]). Although we expect similarities with marine wracks, the composition and mechanisms of formation of inland lake wracks have not yet been described in the literature.

Wrack accumulations on marine coasts and along river banks are of great ecological importance (overview: [6]). Submerged deposits of driftwood (coarse woody debris, CWD) are structural elements that stabilise the beach and encourage the deposition of seston [46]. CWD increases spatial habitat heterogeneity [47, 48], providing both protection for juvenile fish from predators and suitable spawning habitats for adult fish [49–53]. Driftwood and accompanying trapped plant debris play an important role in the dispersal of plant diaspores and animals [38, 48, 54–56]. Wrack accumulations from the remains of submerged plants are a locally significant source of degradable organic matter and nutrients on marine beaches [57–59], which gets washed into the underlying sediment after microbial mineralisation [60–62]. Interspersed with microorganisms, wrack forms the nutritional basis for detritivorous animals [38, 63, 64], which in turn are at the base of the littoral food web (e. g. ground beetles, spiders: [38, 65–69], waders and insectivorous birds: [70–75]). Due to their high nutrient availability [76], aged marine wrack lines often support a particular vegetation (FFH-Habitat Type 1210 Annual Vegetation of Drift Lines [77]).

It is likely that wrack accumulations in inland lakes also contribute similar ecological benefits to the surrounding ecosystem. However, our current understanding of wrack’s ecological significance is mostly based on studies on seashores and river banks, with very few studies focusing on lakeshores (e.g. [42]), except for studies on snags and woody debris (overview: [78]).

In this study, we investigated for the first time the spatial distribution, modes of formation and composition of 36 wrack lines in a large Alpine lake, Lake Constance-Obersee, in March 2019 and March 2020. Using partial least squares (PLS) regressions and multivariate comparisons of paired samples (Hotelling’s *T*^*2*^), we determined the simultaneous effects of relevant environmental factors on the abundance and composition of wrack lines.

We expected that both the spatial dimensions and the volume of wrack lines increase with high exposure of the shore to wind and ship waves, a wide eulittoral and sublittoral zone, and a coarser sediment surface. Also, the composition of the wrack was assumed to be dominated by lake-borne material classes when the wave exposure and the width of the littoral zone are high.

## Methods

### Study area

The field survey was carried out on the shores of the western part of Lake Constance-Obersee. Lake Constance is the third largest and the second deepest lake in Central Europe [79] with a surface area of 536 km² and a maximum depth of 251 m. It is divided into the shallower, mesotrophic ‘Untersee’ and the deep, oligotrophic ‘Obersee’ (472 km^2^). The total shoreline length of the Obersee is 186 km. The proportion of the littoral area (0–10 m water depth) colonised by submerged macrophytes is 66 km^2^ (14% of the lake surface [79]).

### Study sites

Twenty near-natural shoreline stretches were selected across the western, southern and northern shores of Lake Constance-Obersee (Fig 4A). The field survey was carried out between 26^th^ February and 12^th^ March 2019, and between 16^th^ and 19^th^ March 2020, before the beach clean-up by local residents and municipalities had started. 17 sections were surveyed in 2019, and 19 sections in 2020. 16 sections overlapped for both years.

The selected shore sections covered a wide range of environmental conditions that occur at Lake Constance-Obersee, but had no major anthropogenic structures such as retaining walls or rip-raps (see S1.1 Table in S1 Data). 19 sites were situated on long and evenly shaped shores. Only one site was located at the mouth of a small stream, which was additionally enriched by foliage from surrounding woodland.

17 sites were exposed to the south and west-southwest (180–248°), and thus exposed to the dominant wind direction (230–290°). Only three sites were exposed to the northeast. The relief in the sublittoral zone was slightly sloping with 0.5° - 3.2°, corresponding to a width between 53 m and 820 m.

Position data in this study use ETRS89 UTM 32N as the coordinate reference system. Elevation data are given either in the reference system DHHN92 (m NHN, Normalhöhennull, see S1 Annex) or relative to the long-term mean water level of Lake Constance (395.24 m NHN, climate normal period 1^st^ Dec 1990 – 30^th^ Nov 2020).

No permits were required to enter the study areas since the sites were on public land outside of nature conservation areas.

### Water level

**Fig 1:**
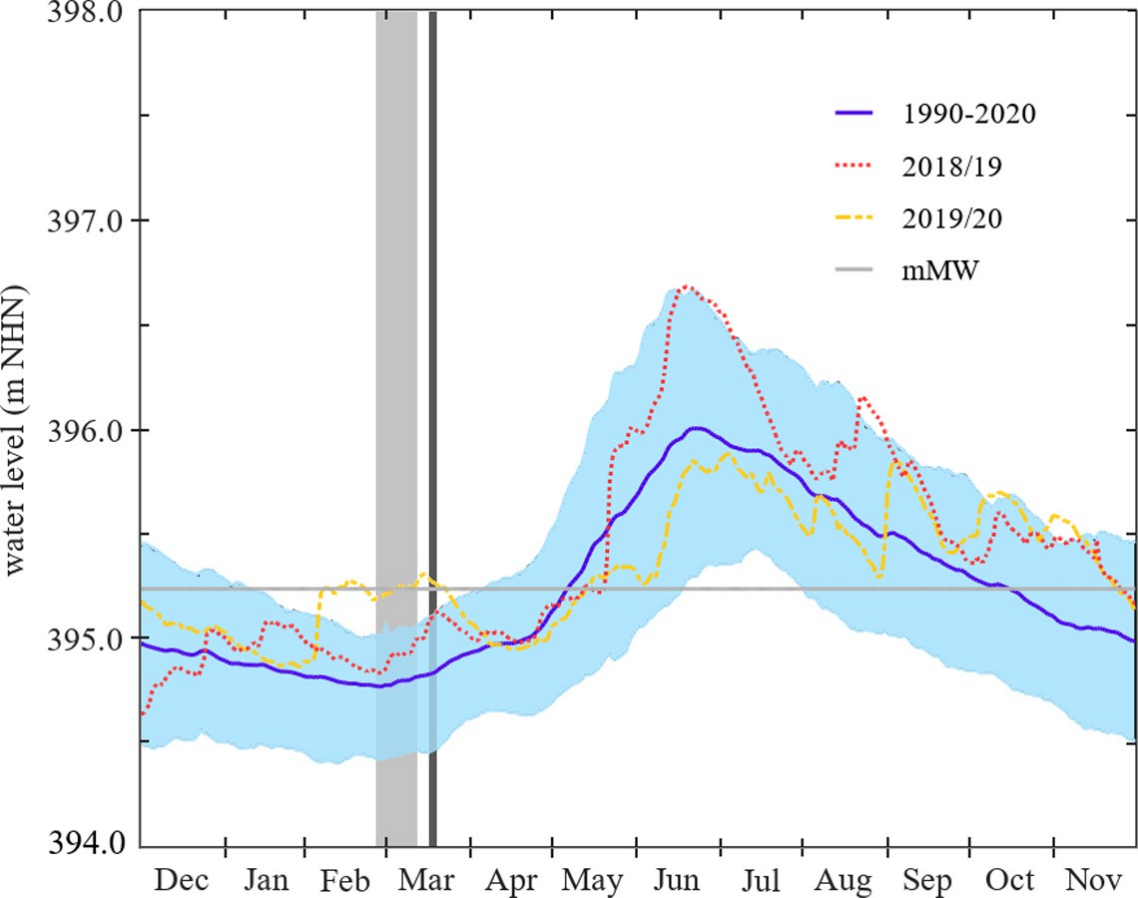
Lake level course of Lake Constance-Obersee. Daily records (m a.s.l., German Datum m NHN) in 2018/19 (red dotted line) and 2019/20 (yellow dotted line), arithmetic mean of daily records in the climate normal period (1^st^ Dec 1990 - 30^th^ Nov 2020, blue line). 10-year exceedance and undercut values (light blue area; calculated on the basis of a logarithmic distribution model) in the climate normal period. The average mean water level (*mMW*) is indicated by a grey line. Source of water level data: Landesanstalt für Umwelt Baden-Württemberg (State Institute for the Environment Baden-Württemberg). The periods of the field studies are highlighted in light grey (2019) and dark grey (2020).

Due to the nival-glacial discharge regime of the Alpenrhein river, Lake Constance is subject to seasonal water level fluctuations. In February-March, the lake level drops to the lowermost annual water level whereas the peak water level is reached at the end of June (Fig 1). The study periods (1^st^ Jan - 31^th^ Mar 2019 and 2020) deviated slightly from the mean value of the normal period, but were within the usual range of variation (80% interdecil of the climate normal period 1^st^ Dec 1990 - 30^th^ Nov 2020).

### Wind

**Fig 2:**
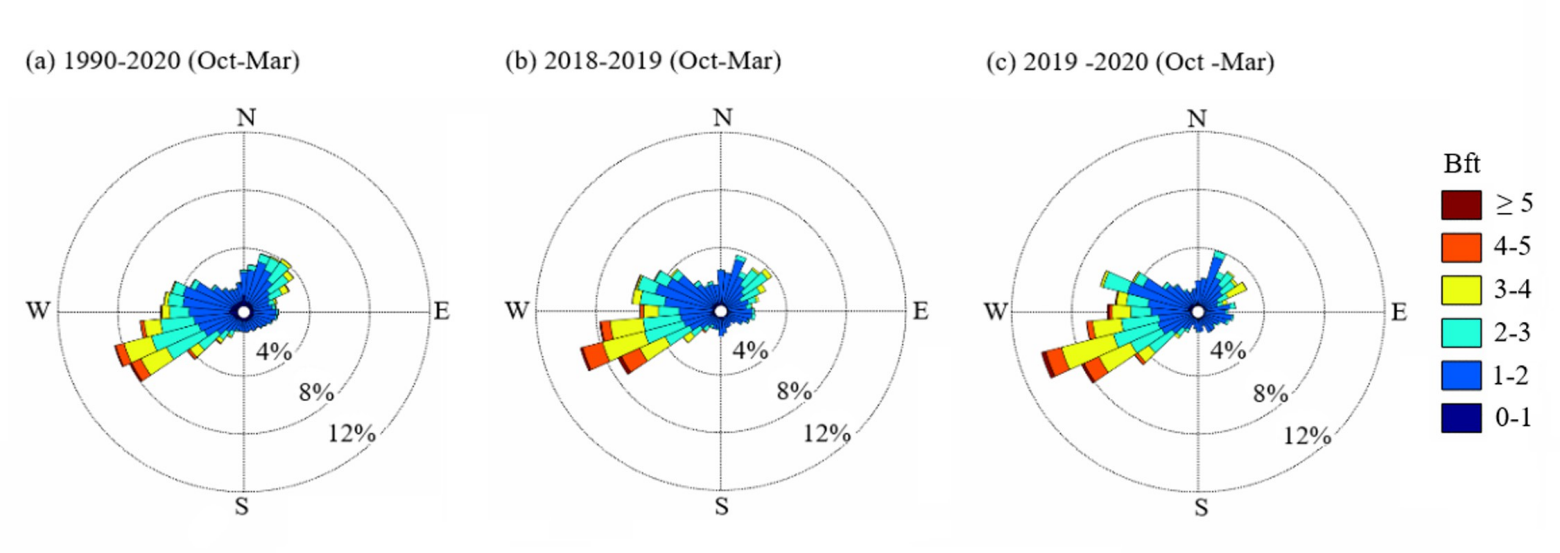
Frequency of winds (36-part wind rose) of the indicated wind force (Bft) in the western Obersee area in the winter half-year (1^st^ Oct - 31^st^ Mar, hourly records). (a) climate normal period 1990/2020, (b) study period 1^st^ Oct 2018 - 31^th^ Mar 2019, (c) 01^st^ Oct 2019 - 31^st^ Mar 2020. Data source: German Meteorological Service DWD, Konstanz weather station ID 2712, UTM 32T 509806.9 5282432.1.

Lake Constance lies in the temperate oceanic climate zone (Cfb) according to the Köppen-Geiger classification [80], in which westerly winds dominate. In the winter half-year (1^st^ Oct - 31^st^ Mar) of the climate normal period, winds from 230 to 290° (42.1% of all hourly records, across all wind strength classes) and 20 to 50° (15.4%) were the most common winds in the western Obersee area (Fig 2A). Winds with 3 or greater Beaufort (Bft, ≥ 3.4 m s^-1^) accounted for 19.2% of the hourly records. These strong winds came predominantly from west-south-westerly directions (230–270°).

During the two study periods, the distribution of wind forces and directions from 1^st^ Oct to 31^th^ Mar was similar to the climate normal period (Fig 2B and 2C). The proportion of west-southwesterly (230–290°) winds (> 0 Bft) was 43.3% in 2019, and 47.4% in 2020. The second most frequent wind direction was north-northeast, again similar to the normal climate period. The proportion of north-northeasterly winds (20–50°) was 13.3% in 2019, and 12.5% in 2020. Winds with 3 or greater Beaufort accounted for 22.9% and 26.0% of the hourly records in 2019 and 2020, respectively. The mean wind speed during the study periods (1^st^ Oct - 31^th^ Mar) was 2.5 m s^-1^ in 2019 and 2.6 m s^-1^ in 2020 (2 Bft). The maximum wind speed was 12.1 m s^-1^ (6 Bft) in 2019 and 14 m s^-1^ in 2020 (7 Bft).

### Surface waves

#### Wind waves

**Fig 3:**
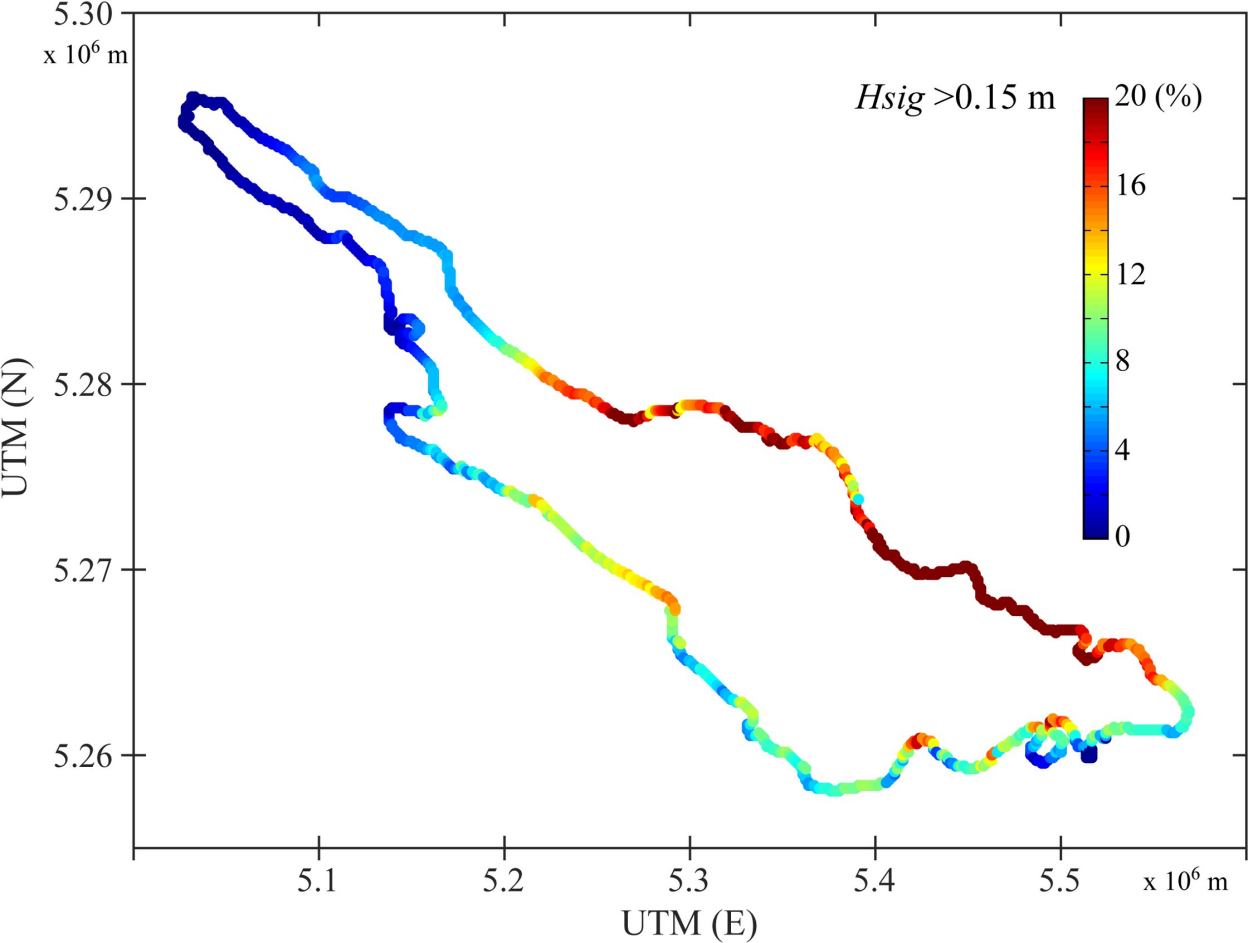
Modelled spatial distribution of the annual-mean wind wave load along the shores of Lake Constance-Obersee (adapted from [82]). The wind wave load along the shores is expressed as the relative frequency of the significant wave height *Hsig* > 0.15 m from Feb 2009 to Jan 2010.

Generally, high wind waves occur sporadically, e.g. triggered by storm events. In Lake Constance, storm events last between a few hours and 1–3 days, with varying magnitudes. During the winter half-year, wind events are more frequent than during summer. Wind speed, direction and effective fetch length determine site-specific wave properties and wave exposure (Fig 3). Wind from storm events causes relatively short wind waves (as compared to ship waves), with wave lengths of 6–10 m (max. 14 m), wave periods of 2–2.5 (3) s, and wave heights of 0.5–1.2 (2) m [81].

To investigate the effect of wind waves on wrack line formation, we used lake-wide simulations of the wind-generated surface wave field and the derived wave exposure around the shores of Lake Constance-Obersee, from February 2009 to January 2010 (simulation period), using the numerical model SWAN (Simulating Waves Nearshore, TU Delft/Deltares, NL). All details about the model setup, parametrisation and follow up analysis of the data are outlined in [82]. The wind wave load along the shores was expressed as the relative frequency of the significant wave height (*Hsig*) > 0.15 m for the simulation period (Fig 3). Wave heights > 0.15 m cause sediment resuspension in the shallow (1 m water depth) nearshore zone, and thus support the formation of wrack. The model results showed that the wind wave load is highly variable in time and space, and is affected by the site-specific effective fetch length in combination with the dominant westerly and north-easterly winds (Fig 2A). The wave load was low (*Hsig* > 0.15 m in 2–6% of the time) in the narrow, northern arm of Lake Constance-Obersee (Überlinger See) and particularly high (10–30% of the time) along the northern shores (Fig 3 and S1.2 Table in S1 Data).

#### Ship waves

**Fig 4:**
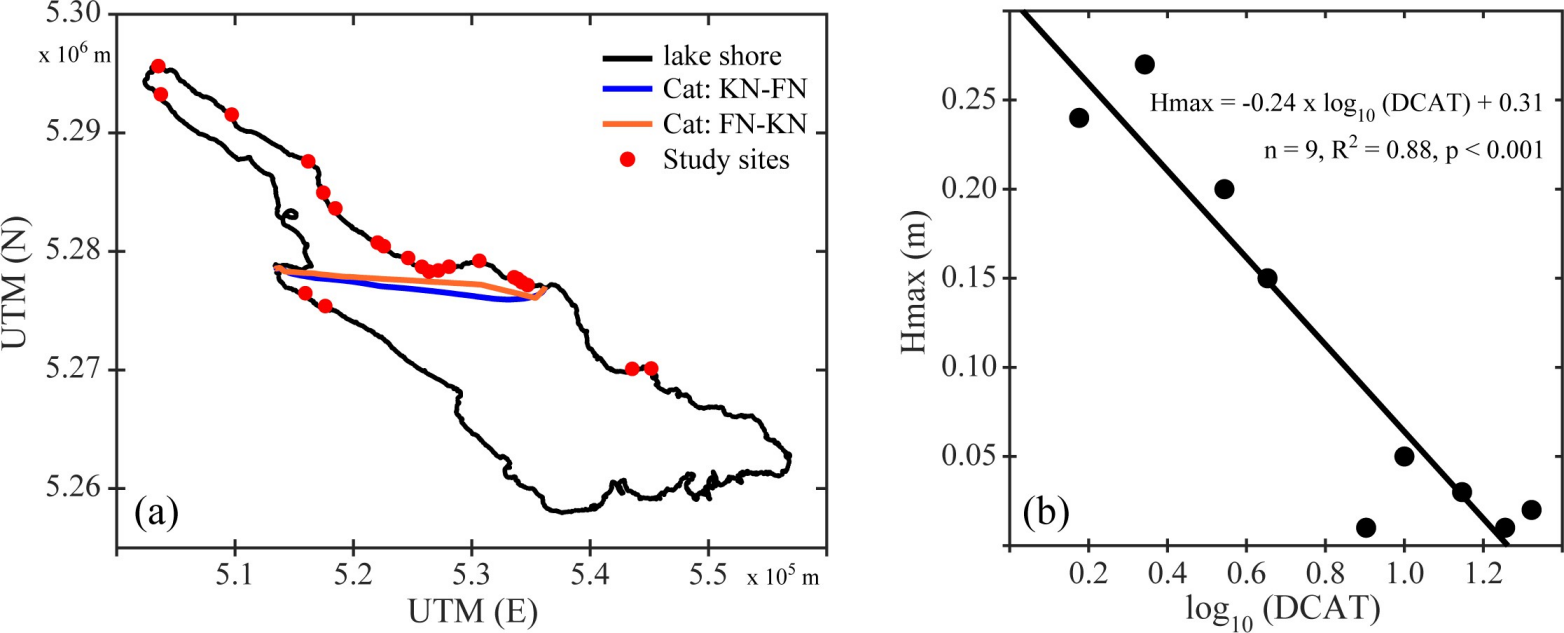
Catamaran tracks and properties on Lake Constance-Obersee. (a) Catamaran cruise tracks between Konstanz (KN) and Friedrichshafen (FN, blue and red line) and the location of the studied shore sections (green dots). (b) Relation between log_10_ (*DCAT* [km]) of the mean catamaran cruise track and measured maximum catamaran wave height at shore sections around Lake Constance-Obersee. The black line represents the linear fit.

Additional waves (ship waves) are created from public transportation across Lake Constance. A fleet of 70 passenger ships, 9 car and passenger ferries as well as 3 catamaran ferries operate on Lake Constance routinely in regular services or charter traffic [83]. In the winter half-year, the ship wave load comes mainly from catamaran ferries between Konstanz and Friedrichshafen, with some contribution from car and passenger ferries between Konstanz and Meersburg, and Romanshorn and Friedrichshafen. The catamarans run at hourly intervals. In the winter season, 2507 passages took place across 182 days (from 1^st^ Oct - 31^st^ Mar) in both directions, corresponding to approximately 2173 hours of operating time. On the open lake, the average cruising speed in both directions was 32.5 km h^-1^. In the bay of Konstanz, the catamarans left at around 23.5 km h^-1^ and entered at around 26 km h^-1^ (Ostendorp, unpubl. data). Catamarans generate unique waves that differ from both wind waves (as described above) and other ship waves. Catamaran waves have longer wave lengths (30–40 m) and wave periods (5–6 s) [84], with induced wave heights between 0.2 and 0.4 m. The divergent catamaran waves propagate from the cruise line (Fig 4A) across the lake with the wave period being maintained or slightly increasing, but the wave height decreasing linearly with the log distance (Fig 4B). In distances of less than 5 km away from the cruise track, catamaran waves cause wave loads that create resuspension of particles in the shallow near-shore zone, which is key to wrack line formation. The minimum distance to the catamaran route (*DCAT*) was used as an indicator of the exposure to catamaran waves.

### Submerged aquatic vegetation and driftwood

The emergent vegetation of pristine shores in Lake Constance-Obersee consists of helophyte beds (mainly *Phragmites australis* (Cav.) Trin. ex Steud. and *Phalaris arundinacea* L.), species-rich low vegetation (*Agrostis stolonifera* L., *Juncus* spp., *Carex* spp., *Littorella uniflora* (L.) Asch. and others) and riverine woodlands (*Salix alba* L., *S*. *fragilis* L., *Quercus robur* L., and others).

The submerged aquatic vegetation of the sublittoral zone extends between the mean annual low water line (*LW*, 394.58 m NHN, 0.66 m below mean water level, *MW*; Ostendorp, unpubl. data), and about 8 m below the *MW* (387 m NHN [85]). The littoral platform (depth zone 0–2 m below *MW*) is dominated by stoneworts (*Chara* spp.) followed by pondweeds (*Stuckenia*, *Potamogeton* spp.). Stoneworts reach a height of 0.05 to 0.3 m and the pondweed stands grow up to the water surface (reaching > 4 m in height). The biomass of closed stonewort meadows ranges between about 0.1 and 0.3 kg m^-2^ ash-free dry matter [86, 87]. With the onset of winter, the pondweeds and stonewort algae largely decay and are washed to the shore during strong winds. Since about 2005, it has been observed that the populations of some stonewort species (*Ch*. *contraria* A. Braun ex Kütz., *Ch*. *globularis* Thuill.) remain until the following spring (K. Schmieder, pers. comm.), so that source material is still available for wrack formation even in late winter.

Under normal weather and water-level conditions, the shores of the western part of Lake Constance-Obersee are only slightly affected by driftwood. The driftwood results from wind breakage of the riverine trees, and from logs coming into the lake via Alpine tributaries.

### Field survey

**Fig 5:**
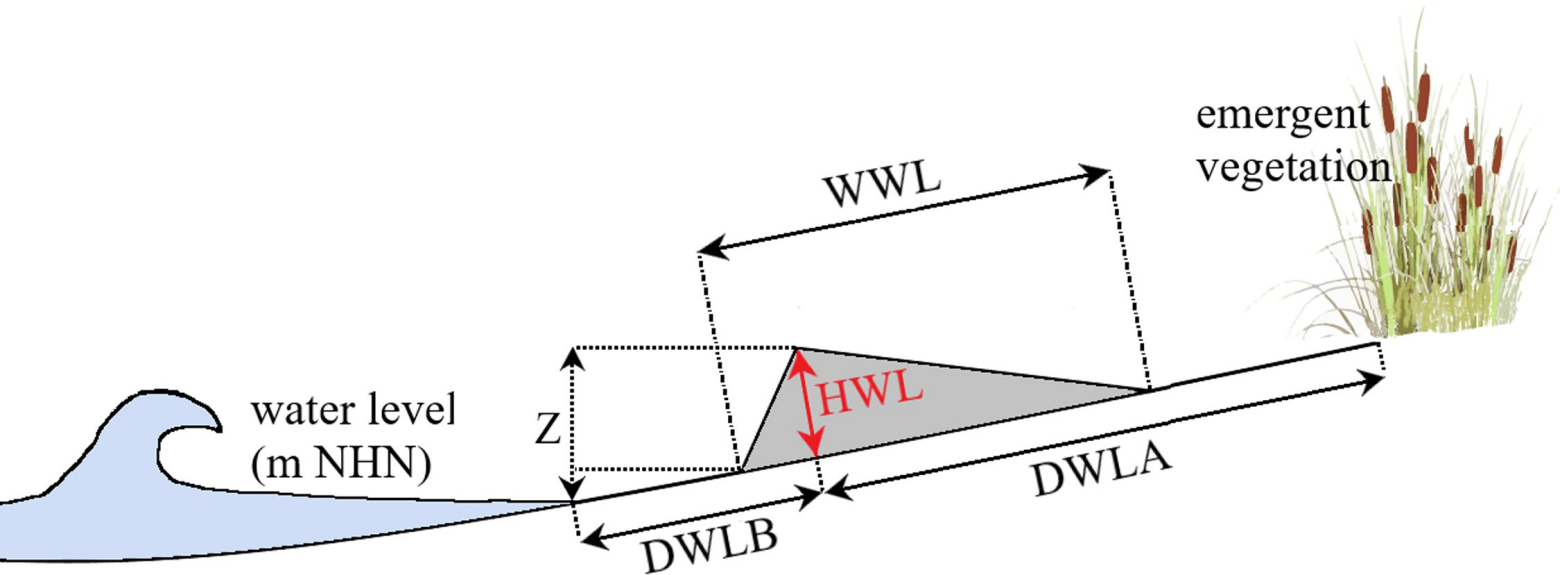
Schematic illustration of the measured variables. *DWLA*–distance of the crest (highest point of the wrack line) to the onset of emergent vegetation, *DWLB*–distance of the crest to the water level, *HWL*–crest height (thickness), *WWL*–average width, *Z*–relative level of the crest above the current lake level (s. text for explanations).

On each shore section, the number of wrack lines (*NWL*) lying behind each other at different elevations on the beach was determined. The following features were measured per wrack line (Fig 5):

- shore-parallel length (*LWL* [m]),
- average width (*WWL* [m]),
- distance of the crest (highest point of the wrack line) to the onset of emergent vegetation (*DWLA* [m]) and the distance of the crest to the water level (*DWLB* [m]),
- average crest height above the ground (thickness) (*HWL* [m]),
- relative level of the crest above the current lake level (*Z* [m]): using the current gauge value and the gauge zero value at gauge Konstanz-Hafen, the absolute level of the crest (*ZWL*_*top*_ [m NHN]) and the base level (*ZWL*_*base*_ [m NHN]) were calculated (*ZWL*_*base*_ = *ZWL*_*top*_—*HWL*),
- percentage composition of the wrack material, assessed visually and aided with graphic reference panels. 20 material classes (*MC*) were distinguished (S1.4 Table in S1 Data).

The specific volume of the wrack (*VWL* [m^3^]) was calculated as the cross-sectional area multiplied by 1 m length of the wrack line. The cross-sectional area was calculated as a triangle using *WWL* und *HWL*. *Vtotal* is the cumulative volume of all wrack lines of one shore section.

Abbreviations, symbols, units and brief definitions of the measured variables are compiled in the S1 Annex.

### Environmental parameters

The following variables for the wrack environment were calculated (S1.2 Table in S1 Data and S1 Methods):

- Shoreline exposure (*ES* [°]): directional angle perpendicular to the shoreline along a stretch of approx. 200 m length.
- Width of the lower eulittoral zone (*WEU* [m]): mean shore-normal distance between the average mean water line (*MW*, 395.24 m NHN) and the mean annual low water line (*LW*, 394.58 m NHN). The inclination of the eulittoral zone is tan α = (395.24–394.58)/*WEU*. The bathymetric data were taken from the digital elevation model of Lake Constance with a grid spacing of 3 m [88].
- Width of the sublittoral zone (*WSUB* [m]): mean shore normal distance between the *LW* line and the 390.50 m NHN isohypse, i.e. the top edge of the deep basin slope, roughly equivalent to the wave base. The inclination of the sublittoral is tan α = (394.58–390.50)/*WSUB*.
- Total effective fetch (*TEF* [m]): the sum of free wind paths over the lake surface between point P_0_ and all points P_E_(α). Calculation method: P_0_ is the point of interest for which the fetch is calculated; this point is by definition at the 4 m depth contour line (391 m NHN), as close as possible to the study site. P_E_(α) is an end point, i.e. the intersection of a beam emanating from P_0_ with the direction angle α with the 4 m depth line on the opposite side of the lake. The fetch *F*(α) is the distance [P_0_ P_E_(α)]. The effective fetch (*EF* [m]) of the direction angle α takes into account not only the main direction (α), but also the beams α-10° and α+10° [89, 90], and is calculated as the mean value. The total effective fetch (*TEF*) is the sum of all effective fetch lengths *EF*(α) over the 36-part compass rose (α = 0°, 10°, …, 350°) (see S1 Methods).
- Total wind exposure of a shore section (*TWE*, *TWE’*, *TWE”*): the sum of all wind exposures *WE*(α) over all angular segments of the 36-part wind rose. *WE*(α) is the effective fetch *EF*(α) weighted with the product of the classified wind force classes (Bft) and the relative frequency of these wind force classes in a given time interval. The wind measurements were from the winter half-year of the study years (1^st^ Oct - 31^th^ Mar 2018/19 and 2019/20). Three variants were calculated: (i) *TWE*–wind data from the Konstanz weather station, which proved to best represent the other six stations around the lake (highest mean correlation values); (ii) *TWE’*–wind data from the weather station closest to the respective shore section, either on the north or south shore of Lake Constance-Obersee; (iii) TWE”–wind data corresponding to the mean values from seven local weather stations, weighted with the reciprocal of the squared distance (1/*d*^2^) between the respective weather station and the shore section in view. All three variants used one of three wind force classes: ≥0 Bft (*TWE0*, *TWE’0*, *TWE”0*), ≥3 Bft (*TWE3*, *TWE’3*, *TWE”3*) or ≥5 Bft (*TWE5*, *TWE’5*, *TWE”5*), resulting in nine variants of the total wind exposure (see S1 Methods).
- Wind wave exposure (*WWEH* [%]): percentage of time the significant wave height *Hsig* exceeds a certain value, derived from the analysis of long-term (1 year), basin-wide wave field simulations using the wave model SWAN (Simulating Waves Nearshore, TU Delft and Deltares). Standard wave parameters, e.g. *Hsig* [m] and *Tsig* [s] (significant wave period) were output every 30 minutes over the entire model period. Model validation is documented in [82]. Ship waves were not considered in the model. The spatial representation of *WWEH* was based on a pre-calculated 150 x 150 m grid with corresponding boundary cells in the vicinity of the 4 m depth contour line. *WWEH* is defined as the percentage of time intervals (3 minutes) in which *Hsig* exceeds the threshold value *Hsig*_,*th*_ (*Hsig*_,*th*_ = 0.10, 0.15, …, 0.40 m) in the respective evaluation period (specific month or whole year) of the modelled wave heights in the evaluation grid cell.
- Position of the landward boundary of the submerged macrophyte vegetation (*UDL*) where the total coverage reaches 10% (*UDL10*) or 50% (*UDL50*), and the water depth at this point.
- Ship wave exposure (*DCAT* [km]): the minimum distance between the landward boundary of the underwater vegetation (*UDL10*) at the shore section in view and the average route of the catamaran (5-sec intervals, GPS readings; mean value of the two directions of travel).
- Grain size classes of surface sediment: visual estimation of the percentage of grain size classes *X%*, *G%*, *S%*, and *UT%* (cobble, gravel, sand, silt and clay, Wentworth class notations) in the sediment surface near the landward boundary of the underwater vegetation.

### Study design

We carried out the following analyses:

- identify the events that caused the lowermost wrack line to form;
- testing the difference between study years (*YR*) concerning (i) the wrack morphology: pairwise comparison (*t*-test, Wilcoxon signed rank test), and (ii) the wrack composition: multivariate pairwise comparison of the study years 2019 and 2020 with *n* = 16 shore sections investigated in both years (Hotelling’s *T*^*2*^ for pairwise comparison);
- identify the effect of environmental variables and the study year (predictors, see below) on (i) the wrack morphology (response variables): partial least squares regression (PLS, s. below), and (ii) the wrack composition (response variables): multivariate partial least squares regression.

#### Response and predictor variables

The following response variables were used as indicators for wrack formation (S1.3 Table in S1 Data):

- number of wrack lines in transect perpendicular to the shoreline (*NWL*)
- average crest thickness of the lowermost wrack line (*HWL*)
- average width of the lowermost wrack line (*WWL*)
- specific volume of the lowermost wrack line (*VWL*)
- level of the base of the lowermost wrack line relative to the mean water level (*ZWL*_*base*_)
- specific volume of all wrack lines (*Vtotal*)
- percentages of the most important material classes (*MCxx*) in the lowermost wrack line,

Variables (i)—(vi) were tested separately, variables in group (vii) were tested simultaneously in the partial least squares regression (PLS, s. below).

The original dataset contained 27 predictors, divided into nine groups, each containing one to nine variables.

1. shoreline exposure: ***ES***
2. shore relief: ***WEU*, *WSUB***
3. total effective fetch: ***TEF***
4. total wind exposure: *TWE0*, *TWE3*, *TWE5*, *TWE’0*, ***TWE’3***, *TWE’5*, *TWE”0*, *TWE”3*, *TWE”5*
5. wind wave exposure: *WWE05*, *WWE10*, ***WWE15***, *WWE20*, WWE2*5*, *WWE30*
6. ship wave exposure (i.e. distance to the catamaran route): ***DCAT***
7. sediment texture: *X%*, *G%*, *S%*, *UT%*, and ***XG%*** = *X%* + *G%*, ***SUT%*** = *S%* + *UT%*
8. upper depth limit of the submerged macrophytes vegetation: ***UDL10***, ***UDL50***
9. year of investigation: ***YR***

To achieve approximately equal weighting across all predictor groups for statistical analyses, the variable that best represented the other variables in the specific group was selected. The criterion was that the square of the correlation coefficient *R*^*2*^ of the representative variables with the other variables should be maximised, and the variability of *R*^*2*^ minimised. Thus a total of 12 variables (bold in the list above) were used for statistical evaluation (S1.2 Table in S1 Data).

#### Statistical evaluation and testing

The predictor and response variables were tested for normal distribution (Shapiro-Wilk test). If H_0_ was rejected, the variables were subjected to a normalising transformation (Johnson S_B_ or S_U_ transformation in most cases; [91, 92]). The transformed variables were analysed for collinearity (Pearson correlation coefficient, Kendall’s τ). Transformed variables are denoted using an asterisk (*) from here on. In most cases, transformations resulted in symmetrical distributions that did not deviate significantly from a normal distribution (S4.1 and S4.2 Tables in S1 Results). Exceptions were X%* and UT%*, as these variables contained many zero values. Therefore, the variables XG%* and SUT%* were calculated as the sum of the neighbouring grain size classes X and G, and S and U+T. Also, the material class gravel (*MC22*) could not be satisfactorily transformed.

The transformed percentages of the terrestrial material classes were significantly negatively correlated with some lake-born components (*p*>|*τ*| < 0.045, *n =* 36 in all cases), but sometimes positively correlated with each other (*p*>|*τ*| < 0.02, *n =* 36). Hence, there was some collinearity in the data set.

The simultaneous influence of the environmental variables (predictors) was analysed with partial least squares regressions (PLS, [93]) using the statistical software JMP^®^ (see S2 Methods). This method is advantageous for analysing ecological data sets with:

- both cardinal and categorical variables,
- relatively small number of observations,
- large numbers of highly correlated predictor variables (Xs, environmental variables),
- a non-normal distribution of many predictors,
- a relatively small signal to noise ratio between predictors and responses (Ys, wrack line properties),
- multivariate datasets (simultaneous effects on >1 correlated response variables).

In univariate cases (wrack line morphology), the effect of study period (*YR*) was tested using a Wilcoxon signed rank test (paired samples). In multivariate analyses (wrack composition, *MCxx*), Hotelling’s *T*^*2*^ test for paired samples was carried out using an Excel add-in by C. Zaiontz (Real Statistics Resource Pack Software, Release 7.6 [94]). The Excel resource additionally provided post-hoc tests used here (Bonferroni-corrected individual tests). To control for the effects of pseudoreplication (pooled data from 2019 and 2020), the year of study was read into the PLS regression as an additional predictor variable.

## Results

### Wrack lines

**Fig 6:**
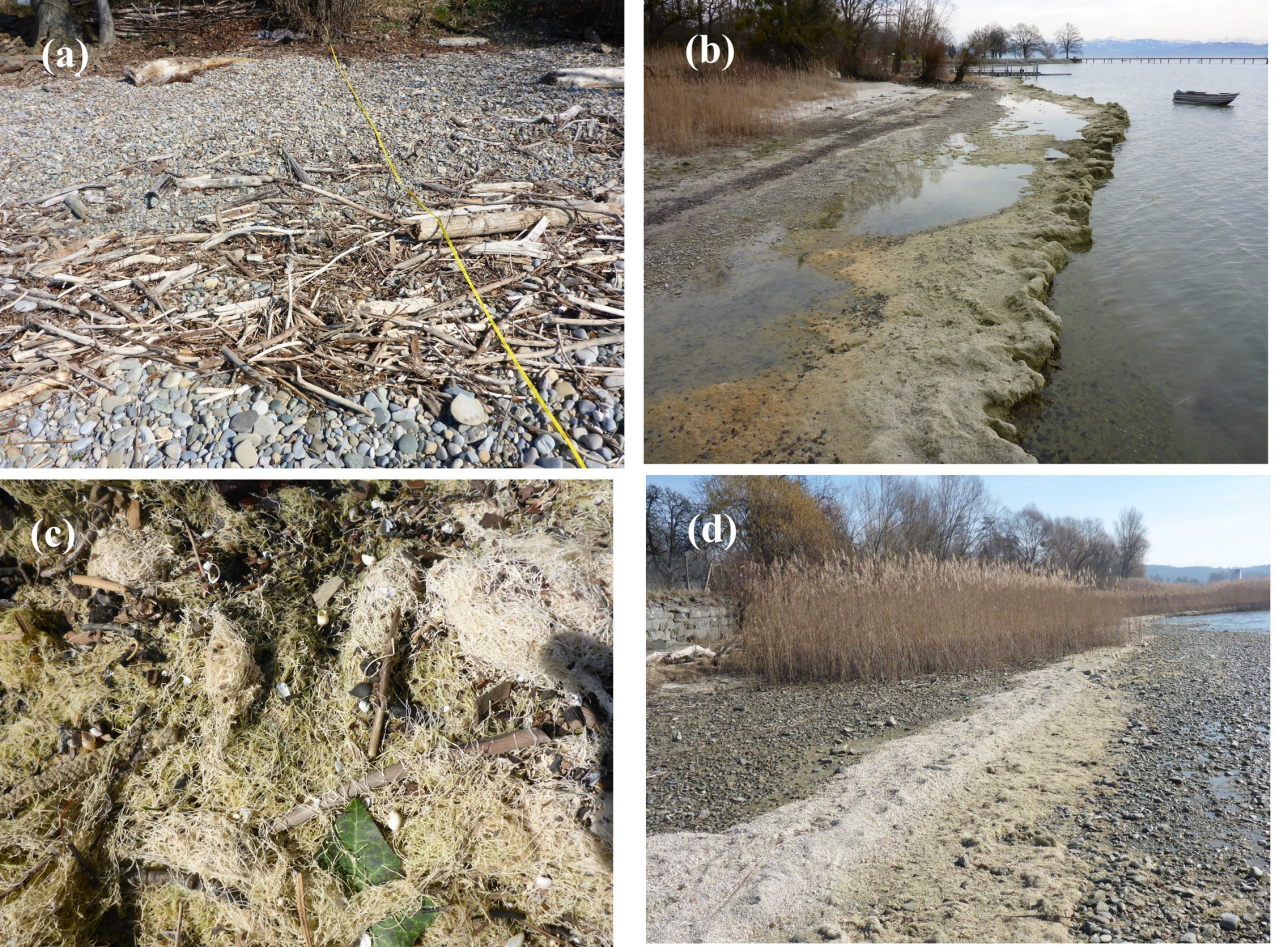
Wrack lines from shore sections of Lake Constance-Obersee. (a) shore section with driftwood (KRTU, 2020), (b) massive wash from *Chara* remnants (FNSB, 2019), (c) surface of a wrack line, with dominant *Chara* remnants (FNFB 2020); (d) wrack line consisting mostly of mollusc shells (*Bithynia tentaculata*, *Dreissena* sp. and others; UMMP 2019). Photos: © W. Ostendorp. See S1.1 Table in S1 Data for location codes.

Of the shore sections surveyed in 2019 (*n* = 17) and 2020 (*n* = 19), all sites had at least one wrack line (Fig 6). The wrack lines extended over long distances (approx. 30 to >300 m). The lowermost wrack line surveyed in March 2020 was particularly pronounced and could be traced almost continuously for several kilometres. It followed the isohypse over long distances, suggesting a uniform formation event.

Most shore sections had only one wrack line. 10 shore sections (2019: 3, 2020: 7) had two wrack lines, and two shore sections had three, differing in location perpendicular to the shore line and elevation above the mean water level. The following evaluations refer to the lowermost wrack line only if not otherwise stated.

The wrack formed shallow wedges that rested on the eulittoral relief. In most cases the lakeside slope was steep, and the landside slope ran gently towards the mineral bed (Fig 6B). The top of the lowermost wrack line (*ZWL*_*top*_) was measured in 2020 only. The elevation was *Md* = 0.32 m (*n* = 16) above the mean water level, corresponding to a lake level of *Md* = 395.55 m NHN. The base of the wrack was *Md* = 0.07 m (*n* = 16) above the mean water level. Most wrack crests were ≤3 m away from the current waterline. The wrack lines were clearly in front of the lakeside boundary of the emergent vegetation, except for one where the ridge of the wrack was within a sparse reed belt. The maximum height (*HWL*) of the lower wrack lines was between 0.03 and 0.70 m, the width (*WWL*) between 0.1 and 4.2 m. The calculated specific volume (*VWL*) varied between 0.002 and 1.23 m^3^ (S1.3 Table in S1 Data).

The wrack material consisted of the following material classes (*MC* [%], see S1.4 Table in S1 Data):

- remains of underwater plants: *Chara* spp. (*MC11*), *Potamogeton/Stuckenia* spp. (*MC12*), *Elodea canadensis* Michx. (MC13), *Myriophyllum spicatum* L. (*MC14*), *Fontinalis antipyretica* Hedw. (*MC15*), filamentous green algae (*MC16*),
- lake-borne mineral components: sand (*MC21*), gravel (*MC22*) and mollusc shells (*MC23*, mainly *Dreissena bugensis* Andrusov and *D*. *polymorpha* (Pallas), as well as *Corbicula fluminea* O.F. Müller and various snail species, mainly *Bithynia tentaculata* L.),
- fragments of *Phragmites australis* (*MC33*, leaves, culms), foliage (*MC31*) and branch fragments of riparian woody plants (*MC32*) and other fragments of terrestrial plants (fruits, seeds, *MC34*, *MC35*),
- anthropogenic wastes such as plastics (*MC45*), glass (*MC42*), brick rubble (*MC41*), charcoal (*MC46*), aluminium (*MC43*) and iron parts (*MC44*).

**Fig 7:**
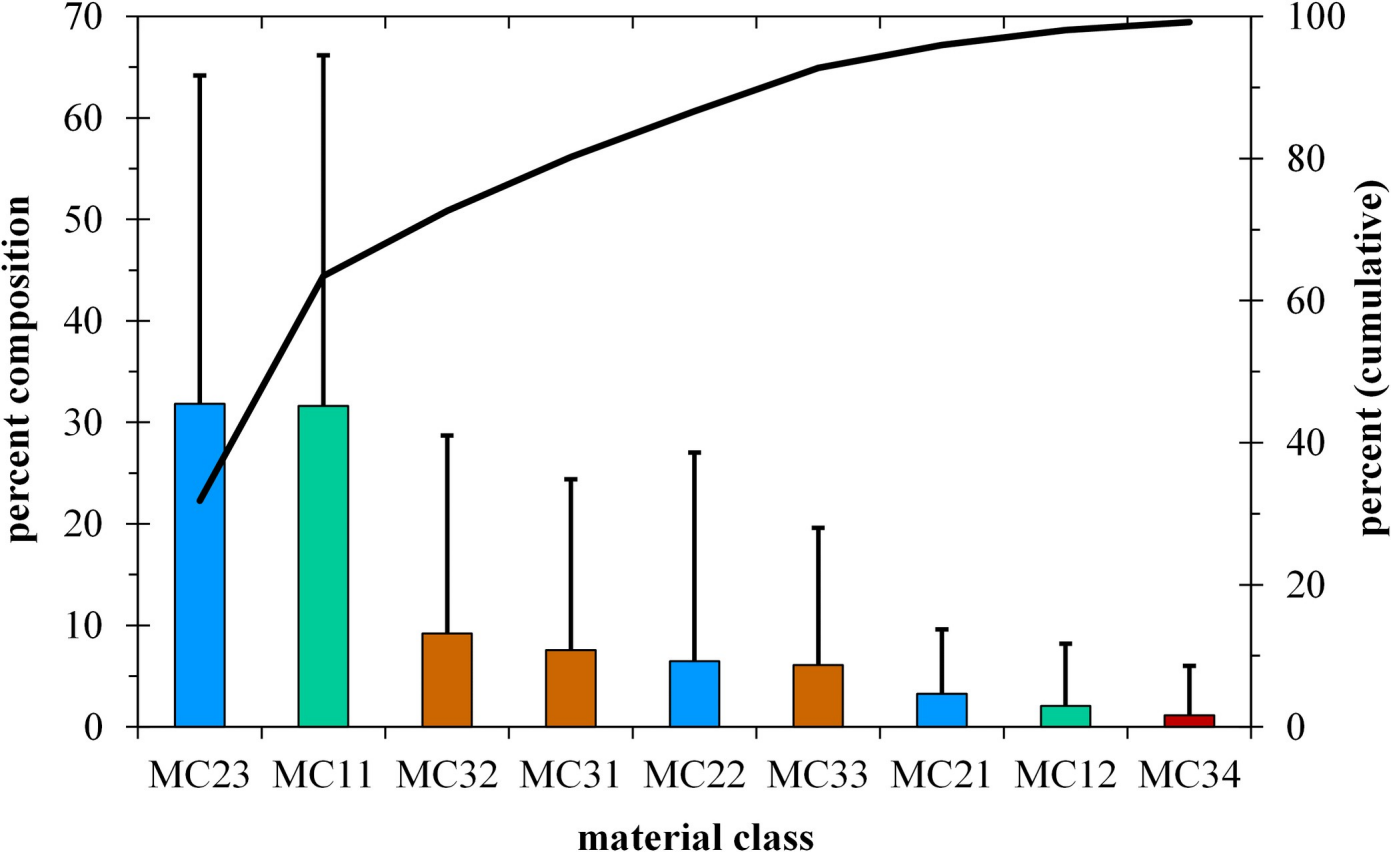
Composition of the lowermost wrack lines. Mean values and standard deviations per material class (*MC* [%]) with on average more than 1% per volume (estimated) of the 36 shore stretches (data from 2019 and 2020 pooled). Black line–cumulative percentage, lake-born components in blue and green, terrestrial components in brown.

However, only six material classes contributed to more than 95% of the volume (Fig 7). The wrack material was dominated by *Chara* remains (*M* = 32%) and mollusc shells (*M* = 32%). Altogether, lake born particles accounted for *M =* 76% of the volume. The most abundant terrestrial particles were foliage and branch fragments. Anthropogenic waste represented *M =* 0.4% of the volume.

### Variation between investigated years 2019 and 2020

Of the 17 study sites investigated in 2019, 16 were also investigated in 2020, allowing a pairwise comparison of the lower wrack line across both years. The water level of Lake Constance-Obersee was significantly higher on the survey dates in 2020 than in 2019 (*M(*Δ) = 0.34 m, *SD(Δ) =* 0.08 m, *p* < 0.001, *n =* 16, Wilcoxon signed rank test; Fig 1).

All measured characteristics did not significantly differ between years (*p* > 0.20 for all morphological variables). The percentages of *Chara* remains and *Potamogeton/Stuckenia* remains were slightly higher in 2020 (*Chara*: *M(*Δ) = 12.6%, *SD(Δ)* = 30.4%, *Potamogeton/Stuckenia*: *M(*Δ) = 3.4%, *SD(Δ) =* 8.3%), the proportion of foliage was lower (*M(*Δ) = -3.5%, *SD(Δ)* = 7.3%). Nonetheless the material composition of the wrack lines did not differ significantly between the two investigation years (Hotelling’s *T*^*2*^ test for paired samples, *T*^*2*^ = 16.0, *F*(6,10) = 1.78, *p*>*F* = 0.20, *n* = 16 pairs).

### Responsible wind events

For estimating the formation period of the lower wrack line, sufficient data was available only for 2020. The period of formation was determined using the following three criteria:

- the lake water level is approximately the same as the mean ground level of the lower wrack line,
- the time window has periods of exceptionally high wind forces,
- these strong winds are directed more or less perpendicular to the shore.

The mean ground level of the lower wrack line in 2020 was 395.34 ± 0.22 m NHN (*M* ± *SD*), i.e. 0.10 ± 0.22 m above the mean water level. Mean shoreline exposure was 210° ± 21° (*M* ± *SD*) for 16 of the 19 shoreline sections sampled in 2020. The remaining three shoreline sections were exposed to the NE (18° - 63°).

**Fig 8:**
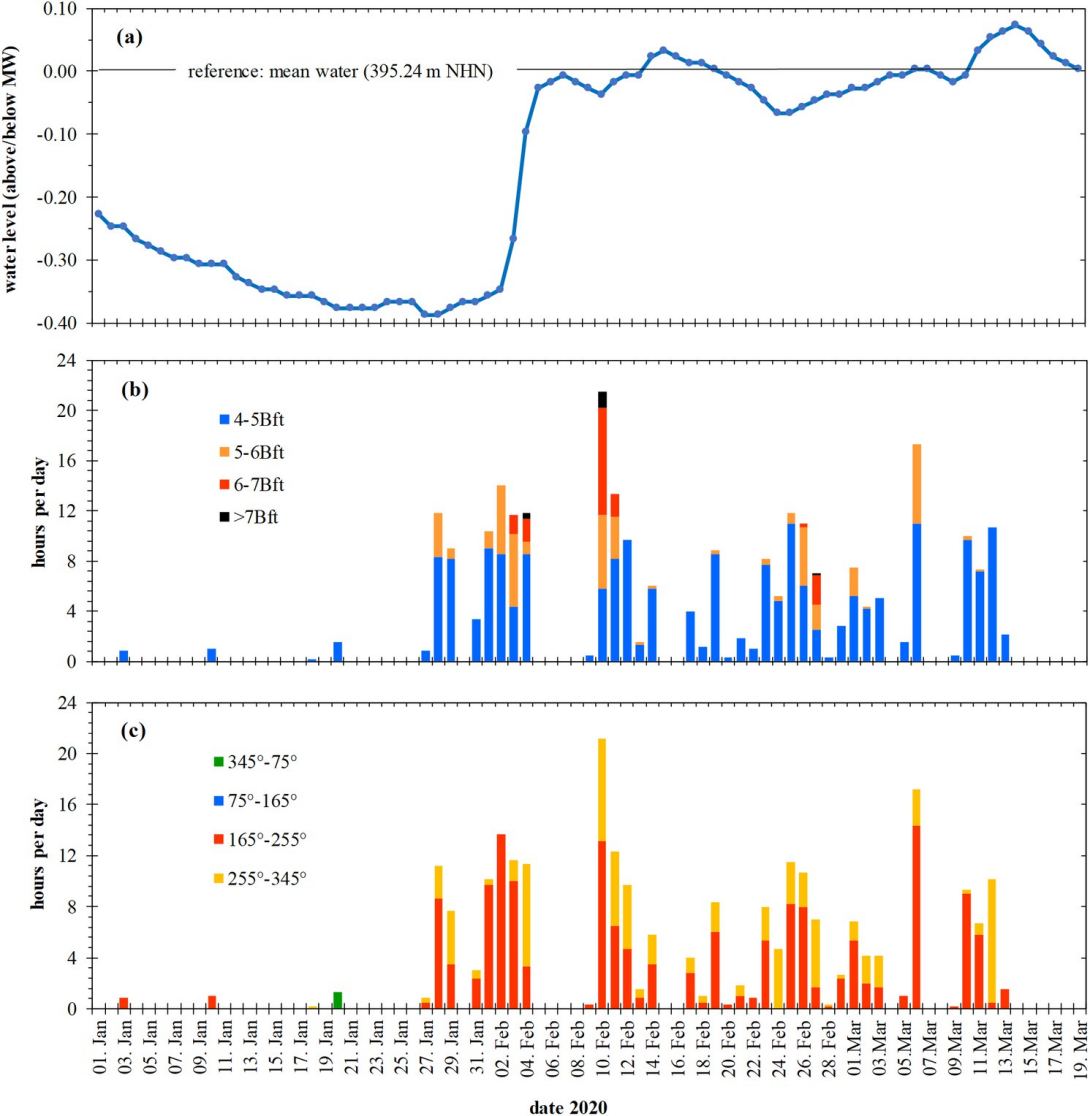
Period of formation of the lower wrack line (January to March 2020). (a) water level (daily mean values, gauge Konstanz-Hafen), relative to the mean water level (period 1^st^ Dec 1990 to 30^th^ Nov 2020). (b) frequency of wind strength classes ≥4 Bft (hours per day, weather station Konstanz, six readings per hour). (c) frequency of wind directions of winds with ≥4 Bft (directional angle, wind rose, six readings per hour). Data sources: Landesanstalt für Umwelt Baden-Württemberg (State Institute for the Environment Baden-Württemberg), LUBW; Deutscher Wetterdienst (German Meteorological Service), DWD.

Fig 8A shows continually decreasing water levels for the period from 1^st^ Jan to 28^th^ Jan 2020. The lake level rose sharply and reached the mean water level very early in the year on 7^th^ Mar. In the following weeks, the water level fluctuated only slightly around the long-term annual mean.

The first weeks of 2020 were characterised by a low-wind period (Fig 8B), followed by a high-wind period from 28^th^ Jan to 13^th^ Mar. The highest wind speeds (max. 15.4 m s^-1^) occurred 10^th^ - 11^th^ Feb at a water level of 0.03 m below mean water level. Winds of 4 to 6 Bft prevailed for 19.9 hours and winds of 6 to 8 Bft for another 11.7 hours within two days.

Winds of ≥4 Bft came mostly from directions between 210° ± 45° (*M* ± *SD*) and reached the northern shore sections on Lake Constance perpendicularly or at an angle of maximum 45° (Fig 8C). Under these conditions, the refraction losses of energy of deep-water waves entering the shallow water zone were low. Winds (and deep water waves) also reached the shore sections from W to NNW (255°-345°) at an acute angle, thus refraction losses in the shallow water zone may have been much higher. The storm event of 10^th^ - 11^th^ Feb brought winds with ≥ 4 Bft blowing perpendicular to the shore, for 13.2 and 6.5 hours, respectively.

Thus, the water level, the duration and preferred direction (165°-255°) of winds ≥4 Bft on 10^th^ and 11^th^ Feb 2020 best fulfil the conditions listed above and therefore best predict wrack formation.

In 2019 the first weeks until 27^th^ Feb were a relatively calm period (8.2% of the time with winds of 4 Bft or stronger). This period was interrupted by two stormy days on 09^th^ and 10^th^ Feb with wind speeds of ≥8 m s^-1^ (5 Bft) over 7.3 hours (max. wind speed 10.6 m s^-1^). The first field survey (five sites on the northern shore of the Überlinger See) was made two weeks later, on 26^th^ Feb. A second monitoring (six sites on the northern shore of the Obersee) was carried out on a calm day after a high-wind period from 28^th^ Feb to 05^th^ Mar with winds of ≥4 Bft in 28.1% of the time (max. wind speed 13.6 m s^-1^, 6 Bft), and the last survey (another six sites at the Obersee) followed after a windy period from 07^th^ to 11^th^ Mar (44.6% winds with ≥4 Bf; max. 11.2 m s^-1^, 6 Bft). Similar to 2020, wind directions of 210° ± 45° prevailed in 2019 (69.8% of time, winds ≥4 Bft), compared to winds from W to NNW (31.2%). Moreover, the water level was low, and rose from 0.40 to 0.25 m below MW from 28^th^ Feb to 12^th^ Mar.

Hence, in contrast to 2020, the wrack lines have formed as a result of either one high-wind event (five sites) or of two (six sites), or even three consecutive events (six sites). Presumably, the second and the third period were more important than the first event.

### Effect of environmental variables on wrack formation

A total of 12 predictors were input into the PLS models to explain the number of wrack lines (*NWL*), the height (HWL*), the base level above or below the mean water level in 2020 (*ZWL*_base_^*^) and the volume of the lowermost wrack line (VWL*), and the total volume of all consecutive wrack lines (Vtotal*). Each variable was run individually (Table 1).

**Table 1:** Overview of the PLS regression results of six response variables on 12 predictor variables.

		response variables					
		*NWL*	HWL*	WWL*	VWL*	*ZWL*_*base*_ *	Vtotal*
*n* (2 years pooled)		36	36	36	36	14	36
no. of factors		no meaningful model	1	1	1	1	1
no of predictors			3	4	4	4	5
*R* ^*2*^			0.23	0.47	0.40	0.54	0.32
*R* ^*2*^_adj_			0.21	0.45	0.38	0.50	0.30
*p*>|*t*|			0.003	<0.0001	<0.0001	0.003	0.0004
eliminated outliers			0	0	0	2	0
*TEF*	*VIP*			0.916	0.817		0.913
	loading			0.531	0.521		0.494
	$\hat{b}$			0.195	0.162		0.131
	*B*			34 × 10^−6^	2.8 × 10^−6^		2.27 × 10^−6^
WEU*	VIP		0.820	0.875	0.881	0.929	0.858
	loading		0.469	0.361	0.371	-0.532	0.277
	$\hat{b}$		0.169	0.187	0.175	-0.208	0.123
	*B*		0.169	0.186	0.175	-0.234	0.123
WSUB*	*VIP*		1.283	1.098	1.237	1.102	1.251
	loading		0.706	0.546	0.557	-0.485	0.504
	$\hat{b}$		0.264	0.234	0.246	-0.247	0.179
	*B*		0.264	0.234	0.246	-0.270	0.179
XG%*	*VIP*					1,099	
	loading					0.512	
	$\hat{b}$					0.246	
	*B*					0.238	
UDL50*	*VIP*					0.846	
	loading					-0.467	
	$\hat{b}$					-0.190	
	*B*					-0.237	
TWE‘3*	*VIP*		0.825				0.941
	loading		0.531				0.435
	$\hat{b}$		0.170				0.135
	*B*		0.197				0.156
DCAT*	*VIP*			1.091	1.012		0.990
	loading			-0.538	-0.530		-0.485
	$\hat{b}$			-0.233	-0.201		-0.142
	*B*			-0.311	-0.269		-0.189
intercept *a*			0.005	-0.576	-0.479	0.107	-0.381

*NWL*–number of wrack lines, *HWL*, *WWL*, *VWL*, *ZWL*_*base*_−height, width, volume and base level above or below mean water level of the lowermost wrack line, *Vtotal*–total volume of all wrack lines. *TEF–*total effective fetch, *WEU*, *WSUB*–width of the eulittoral and sublittoral zone, *XG%*–percentage of cobbles and pebbles in the surface sediments, *TWE’3* –total wind exposure with reference to winds ≥3 Bft, *UDL50* –upper depth limit of the submerged vegetation, based on 50% coverage, *DCAT*–minimum distance to the route of the catamaran. *n*–sample size, *R*^*2*^, *R*^*2*^_adj_ and *p*>|*t*|–(adjusted) coefficient of determination, probability of error for the correlation of Y_actual_ on Y_predicted_. *VIP*–variable importance for the projection, $\hat{b}$ –regression coefficient of the standardised X in the pruned PLS regression model, *b*–regression coefficient of the original X in the pruned PLS regression model. *a*–intercept in the equation Y = Σ (*b*_i_ × X_i_) + *a*. Normalised transformed variables are highlighted with an asterisk (S4.1 Table in S1 Results). Only those predictor variables that achieved a *VIP* > 0.8 in at least one model are shown.

The number of wrack lines (*NWL*) did not depend on any predictor variable, including *YR*, since the optimal number of latent factors was zero. However, the model run indicated that wind wave exposure (WWE15*) and ship wave exposure (DCAT*) were the most important variables. Multiple linear regression between *NWL* and these predictors led to a significant prediction:

$$\begin{array}{l} NWL=0.0296\;\times \;WWE15*+\;0.314\;\times \;DCAT*+\;0.938;R^{2}=0.19,\\ p>\left|t\right|\;=0.009;\;\text{n}=36 \end{array}$$

However, multiple linear regression does not account for collinearity among predictor variables.

The width, height, volume and base level of the lowermost wrack line, and the total volume of all wrack lines could be successfully modelled from the chosen predictors (Table 1). The year of investigation (*YR*) had no significant effect (*VIP* = 0.01–0.73), and was not included in the pruned models.

The optimised models explained about 23 to 54% of the variance of the response variables, and were highly correlated with the measured data (*p*>|*t*| < 0.005 in all cases).

The most important and influential predictor across all response variables was the width of the sublittoral zone (WSUB*, *VIP* = 1.10–1.28, |$\hat{b}$| = 0.18–0.26), followed by the width of the eulittoral zone (WEU*, *VIP* = 0.82–0.93, |$\hat{b}$| = 0.12–0.21). The width, height, and thus the volume of the lowermost wrack line were positively correlated with the width of the sub- or eulittoral zone. The total volume of all wrack lines was also positively correlated with WSUB*. In comparison, the base level of the lowermost wrack line was negatively correlated with littoral zone width ($\hat{b}$, *b* < 0), i.e. on steep shores, the lower wrack line lay at higher elevations upon the shore than upon gently sloping shore sections.

Sediment texture was a relevant predictor for Vtotal*. More elevated wrack lines were correlated with coarser sediments ($\hat{b}$, *b* > 0). The ground level of lowermost wrack lines was higher on shores with coarse sediment than on those with fine sediment.

Wave exposure also predicted wrack features: with increasing total effective fetch (*TEF*), the width and volume of the lowermost wrack line as well as the total volume of all wrack lines increased. High wind exposure drove thicker wrack lines and a larger total wrack volume. However, the relevance and prediction strength of *TEF* and *TWE’3* were rather low (*VIP* = 0.82–0.94; |$\hat{b}$| = 0.13–0.20). The influence of ship waves (DCAT*) was slightly more significant and negatively correlated with response variables (*VIP* = 0.99–1.09; $\hat{b}$, *b* < 0), i.e., shoreline sections closer to the catamaran route had a wider and more voluminous lowermost wrack line, and the total wrack volume was higher. This effect was strongest for the width of the lowermost wrack line (|$\hat{b}$| = 0.23).

The upper depth limit of submerged macrophytes (UDL50*) only influenced the ground level of the lower wrack line (*ZWL*_*base*_). Sites with a high laying depth limit, i.e. closer to the mean water line, exhibited lower wrack line levels on the shore.

**Fig 9:**
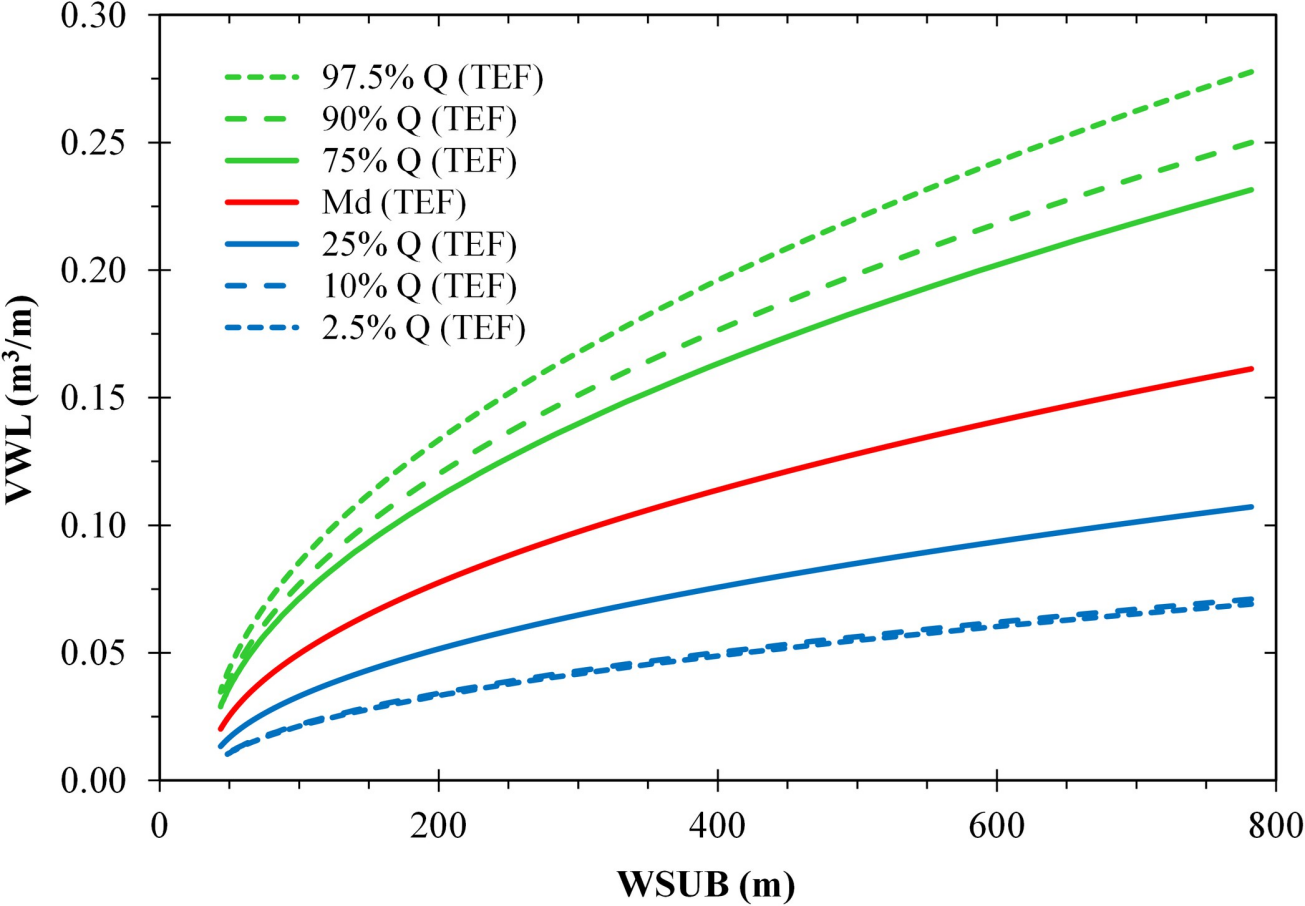
Estimated effects of the width of the sublittoral zone (*WSUB* [m]) and the total effective fetch (*TEF* [m]) on the specific volume of the lowermost wrack line (*VWL* [m^3^ m^-1^]) based on a PLS regression model. *TEF* is represented by sets of curves of the quantiles 97.5% to 2.5%. The other predictors of the model (WEU*, DCAT*, Table 1) were replaced by multiple regressions on WSUB* and TEF*.

**Fig 10:**
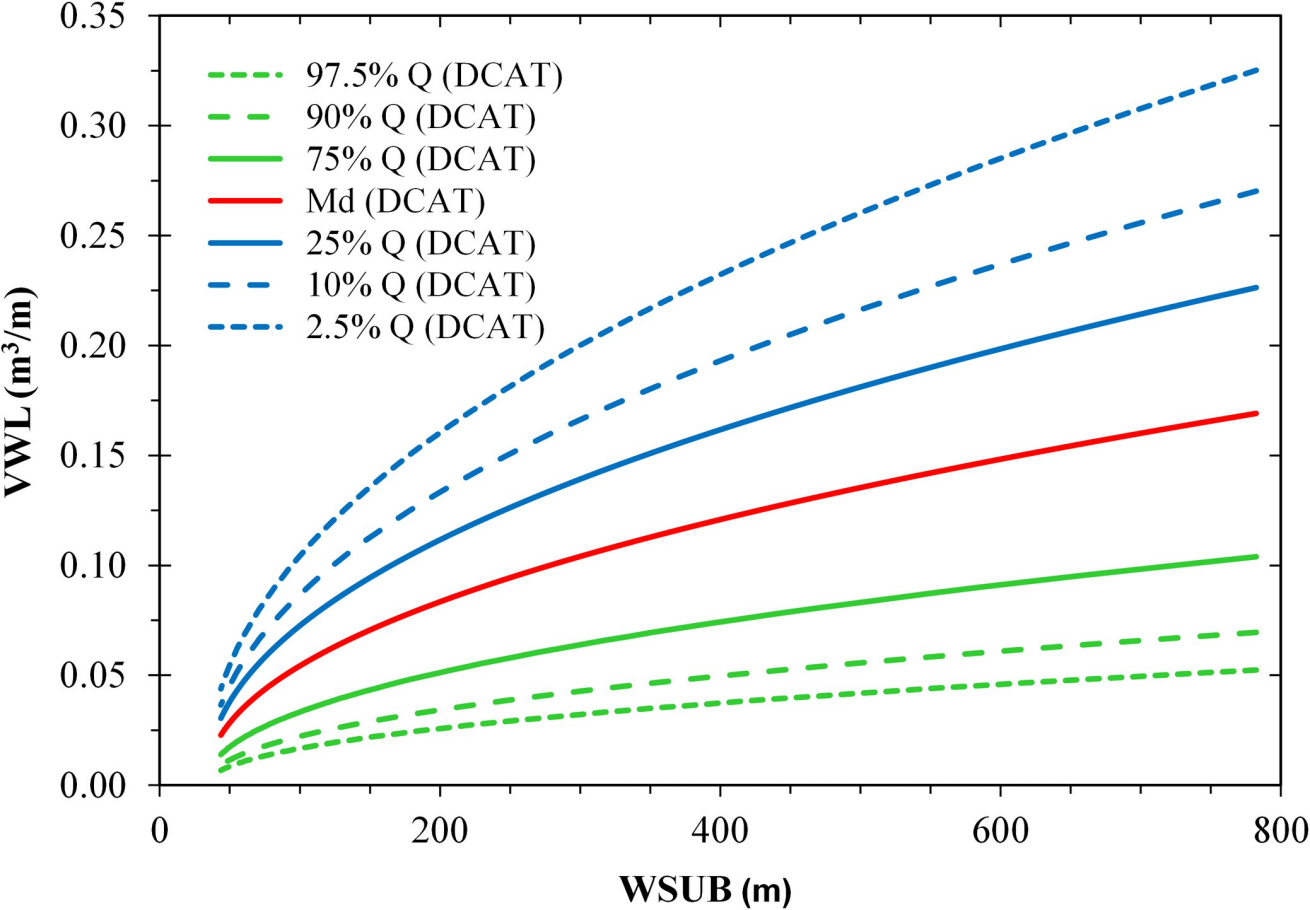
Estimated effects of the width of the sublittoral zone (*WSUB* [m]) and the distance from the catamaran route (*DCAT* [km]) on the specific volume of the lowermost wrack line (*VWL* [m^3^ m^-1^]) based on a PLS regression model. *DCAT* is represented by sets of curves of the quantiles 97.5% to 2.5%. The other predictors of the model (TEF*, WEU*, Table 1) were replaced by multiple regressions on WSUB* and DCAT*.

The modelled influence of the total effective fetch was stronger for wide sublittoral zones (*WSUB* > 200 m) than for narrow ones (*WSUB* < 100 m) (Fig 9). Similarly, the expected influence of the distance to the catamaran route increased with increasing sublittoral width (Fig 10). However, the effect strengths (|$\hat{b}$| = 0.13–0.23) were smaller than the effect strength of *WSUB* (|$\hat{b}$| = 0.18–0.26).

**Fig 11:**
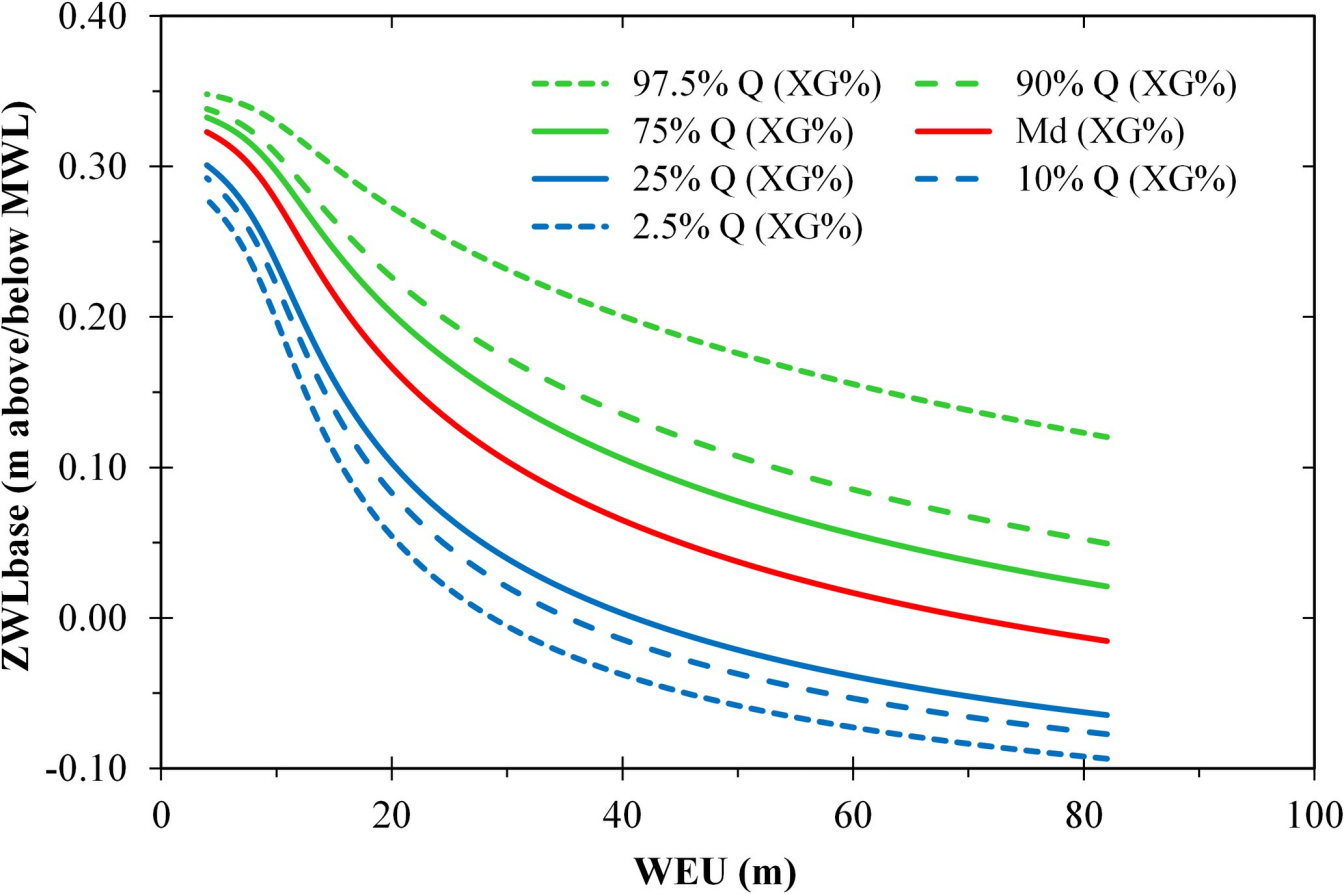
Estimated effects of the width of the lower eulittoral zone (*WEU* [m]) and the surface sediment texture (*XG%* [%]) on the vertical position of the lower wrack line (*ZWL*_*base*_ [m], difference from the mean water level) based on a PLS regression model. *XG%* is represented by the sets of curves of the quantiles 2.5% to 97.5%. The other predictors of the model (WSUB*, UDL50*, Table 1) were replaced by multiple regressions on WEU* and XG%*.

On very narrow shores (*WEU* < 10 m), we found a relatively strong effect of the eulittoral width on the base level of the lowermost wrack line (*ZWL*_*base*_) modified by the sediment texture (Fig 11). On wider beaches (*WEU* > 30 m), a weaker effect of *WEU* and a strong effect of *XG%* on *ZWL*_*base*_ was predicted by the pruned PLS model.

### Effect of environmental variables on the particulate composition of the wrack lines

All 12 predictors were modelled to explain the particle composition of the lower wrack line (*n =* 36) using a multivariate PLS regression model (Table 2).

**Table 2:** Results of the PLS regression of the six most abundant material classes of the lowermost wrack line on 12 predictor variables.

		response variables					
		MC11*	*MC22*	MC23*	MC31*	MC32*	MC33*
n (2 years pooled)		36					
no. of latent factors		2					
explained cumulative X variation		f1: 47.5% f2: 33.6%					
explained cumulative Y variation		f1: 16.1% f2: 10.3%					
no. of predictors		3					
Y loadings		f1: -0.602 f2: -0.113	f1: 0.302 f2: -0.175	f1: -0.324 f2: -0.162	f1: 0.220 f2: 0.507	f1: 0.623 f1: 0.246	f1: 0.066 f2: 0.783
*R* ^*2*^		0.37	0.11	0.12	0.21	0.41	0.38
*R* ^*2*^_adj_		0.35	0.08	0.09	0.18	0.40	0.37
*p*>|*t*|		<0.0001	0.052	0.041	0.005	<0.0001	<0.0001
eliminated outliers		0					
WEU*	*VIP*	1.07					
	loading	f1: -0.698 f2: 0.233					
	* $\hat{b}$ *	0.393	-0.172	0.222	-0.193	-0.421	-0.125
	*B*	0.386	-3.484	0.235	-0.195	-0.393	-0.123
SUT%*	*VIP*	1.05					
	loading	f1: -0.405 f2: 0.849					
	$\hat{b}$	-0.003	-0.171	-0.076	0.346	0.099	0.571
	*B*	-0.0025	-3.453	-0.808	0.348	0.092	0.558
DCAT*	*VIP*	0.94					
	loading	f1: 0.591 f2: 0.473					
	$\hat{b}$	-0.377	0.100	-0.241	0.315	0.439	0.335
	*B*	-0.494	2.705	-0.341	0.424	0.549	0.438
intercept *a*		0.146	6.611	0.048	0.027	-0.138	0.100

*MC11* –% charophyte algae remains, *MC22* –% gravel, *MC23* –% mollusc shells, *MC31* –% dead foliage from riparian trees, *MC32* –% branch material, *MC33* –% reed (*P*. *australis*) stems and leaves, *WEU*–width of the eulittoral zone, *SUT%*–percentage of fine sediments in the sediment surface, *DCAT*–minimum distance to the route of the catamaran. *n*–sample size, *R*^*2*^, *R*^*2*^_adj_ and *p*>|*t*|–(adjusted) coefficient of determination, probability of error for the correlation of Y_actual_ on Y_predicted_. *VIP*–variable importance for the projection, $\hat{b}$ –regression coefficient of the standardised X in the pruned PLS regression model, *b*–regression coefficient of the original X in the pruned PLS regression model, *a*–intercept in the equation Y = Σ (*b*_i_ × X_i_) + *a*. f1, f2 –latent factors in PLS. Normalised transformed variables are indicated with an asterisk (S4.2 Table in S1 Results). Only predictor variables that achieved a *VIP* > 0.9 are shown.

The multivariate PLS regression yielded a stable number of three relevant predictors with *VIP* > 0.9 which were projected onto two latent factors and explained 26.4% of the cumulative variation in the six response variables (Y-variability). The regression model did not contain any outliers. The width of the eulittoral zone (WEU*) and the distance to the catamaran route (DCAT*) were highly correlated with the first factor, and the percentage of fine sediments (SUT%*) was best correlated with the second factor.

The most important response variables were charophyte algae remains (MC11*), branch material (MC32*) and reed stems and leaves (MC33*) which explained between 37% and 41% of the total Y-variability. Gravel (*MC22*), mollusc shells (MC23*) and foliage from trees (MC31*) explained only 11–21% of the total Y-variability. We note that the results for gravel (*MC22*) are weak, as regression showed a very poor fit to the normal distribution (normal-quantile plot) and a low expectation reliability (higher bias) for the residuals.

The relevant predictors of particle composition were shore topography and sediment texture (WEU*, SUT%*) followed by ship wave exposure (DCAT*). These predictors had a highly significant effect (*p>|t|* < 0.0001) on charophyte algae remains, branch material and reed stems and leaves, whereas the influence on gravel and mollusc shells was weak. The effect of the year of investigation (*YR*) was weak in the initial model run (*VIP* = 0.10) so that is was not included in the pruned model.

Broad eulittoral platforms and short distances to the catamaran route favoured a high percentage of charophyte remains (MC11*, |$\hat{b}$| = 0.38–0.39, Table 2), whereas the abundance of branch material (MC32*) was high on narrow eulittoral zones more distant from the catamaran route (|$\hat{b}$| = 0.42–0.44, Table 2). The dead foliage from riparian trees and dead stems (MC31*) and leaves from *Phragmites* reeds (MC33*) were positively influenced by fine sediments in the sublittoral ($\hat{b}$ = 0.35–0.57), and also positively correlated with distance from the catamaran route (DCAT*; $\hat{b}$ = 0.32–0.34). The width of the eulittoral platform had a small negative effect ($\hat{b}$ = -0.13 to -0.19).

**Fig 12:**
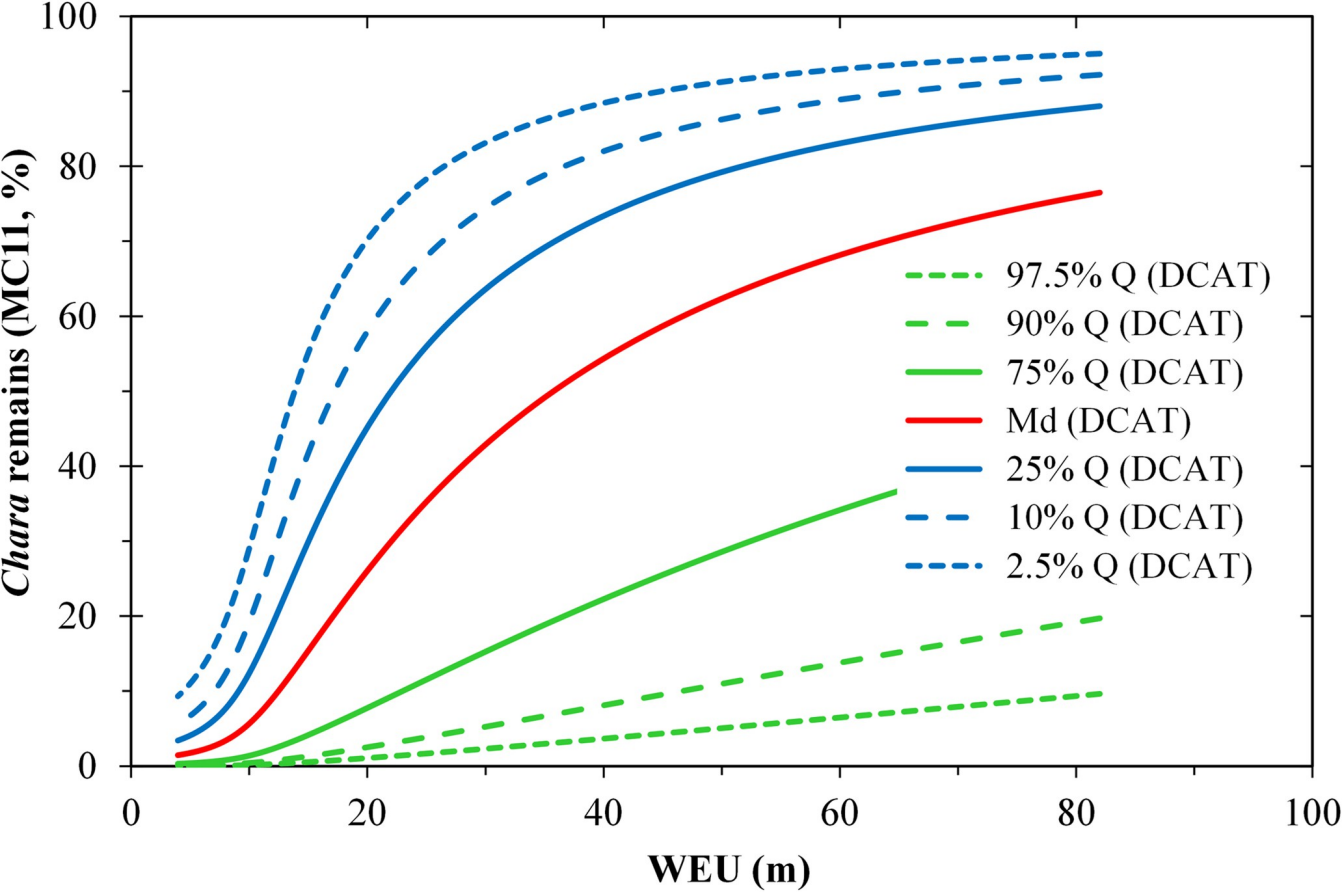
Estimated effects of the width of the lower eulittoral zone (*WEU* [m]) and the distance to the catamaran route (*DCAT* [km]) on the percentage share of *Chara* remains (*MC11* [%]) in the lower wrack line, based on a PLS regression model. *DCAT* is represented by the sets of curves of the quantiles 2.5% to 97.5%. The other predictor in the model (SUT%*, Table 2) was replaced by a multiple regression on WEU* and DCAT*.

At distant shore segments (*DCAT* ≈ 17 km, i.e. the 90% quantile), there is a comparatively small effect of *WEU*, whereas a reduction in distance (*DCAT* ≈ 3 km, i.e. 50% percentile) led to a significant increase in the proportion of *Chara* in the wrack (Fig 12). At shore sections near the catamaran route (*DCAT* < 2 km, i.e. 25% percentile), the PLS regression model suggests a small effect of *DCAT* but a pronounced effect of the eulittoral width, especially in the range between 10 and 40 m. Yet a further increase in eulittoral width to over 80 m had only a minor effect on the *Chara* percentage. In comparison, the terrestrial material classes (*MC31*, *MC32* and *MC33*) behaved reversely, as the sum of the six most frequent material classes was close to 100% (*M =* 93%).

### Summary of tests

A total of seven individual PLS regression models were tested in this study to explain the simultaneous influence of 12 predictor variables on the extent, location, volume and composition of the wrack lines. Six models produced a result with at least two and at most five predictors with *VIP* > 0.8 and |$\hat{b}$| > 0.1), which significantly correlated with the measured response variables (*p>|t|* < 0.005 in most cases; Tables 1 and 2).

Eight of the 12 predictors were represented in at least one pruned (optimised) model (Table 3). The most frequently occurring predictors were (in descending order) *WEU* > *DCAT* > *WSUB*, each of which was represented in five or more of the pruned models. The spatial dimensions and volume of the lower wrack line were best explained by *WEU* ≈ *WSUB* > *DCAT* ≈ *TEF*. The particulate composition was best explained by *WEU* > *DCAT*. The year of study had no significant influence (*VIP* < 0.75 for all response variables).

**Table 3:** Overview of the results of PLS regressions for the position (*ZWL*_base_*), shape (HWL*, WWL*) and volume of the lowermost wrack line (VWL*), the volume of all wrack lines (Vtotal*), and the percentage composition of the wrack (*MCxx*), based on the six most abundant material classes. See Tables 1 and 2 for details.

			predictors							
			conditions in the offshore donor sites				conditions of detachment and transport			conditions in the recipient system
			WSUB*	UDL50*	XG%*	SUT%*	*TEF*	TWE‘3*	DCAT*	WEU*
response	position	*ZWL*_base_^*^	- -	-	++					- -
	shape and volume	HWL*	++					+		+
		WWL*	++				+		- -	+
		VWL*	++				+		- -	+
		Vtotal*	+				+	+	-	+
	composition (6 most abundant MC)	MC11*							- -	++
		*MC22*				-				-
		MC23*							- -	++
		MC31*							++	-
		MC32*							+++	- - -
		MC33*				+++			++	-

+, - − positive, negative sign of the regression coefficient $\hat{b}$ in the pruned PLS regression model for the standardised predictor variable. +, ++, +++, -, - -, - - - − strength of the effect of a predictor variable on the response variable (|$\hat{b}$| >0.1–0.2, >0.2–0.4, >0.4).

## Discussion

Wrack lines are banks of phytodetritus intermixed with driftwood, carcasses of animals, plastics debris and sediments, which extend parallel to the contour lines of vegetation-lacking beaches. They are typical features on marine tidal and non-tidal coasts, as well as estuaries, river banks (e.g. [95]) and shores of large lakes (e.g. [41–45]). In this study, we analyse the abundance, composition and the conditions of wrack formation on the shore of Lake Constance-Obersee, a large oligotrophic Alpine lake with near-natural lake level fluctuations. To the best of our knowledge this is the first detailed study on the factors of wrack formation and composition for an inland lake.

### Conceptual framework

The amount of wrack in a given area of a marine beach face is highly variable [31: p.150, 32: p.71] with large scale variation along the shoreline, small scale variation along the cross-shore profile, and variations along both short (tidal cycle, hours to days) and long timescales (seasons, weeks to years). Wrack lines are dynamic accumulation bodies which undergo resuspension by tides and waves, mechanical breakdown via drying-rewetting cycles, wind-driven displacement along the shoreline, and microbial decomposition and feeding by detritivores [31, 36]. In this study we analyse the variability along the shoreline, and the variability between two consecutive years, but did not survey any seasonal effects or wrack dynamics.

The abundance of wrack can be quantified by visual estimation of cover percentage [39, 96, 97], by measuring its width and height [31], by calculating its volume [40], or by taking representative samples and calculating the wet weight, dry weight or ash-free dry weight [32, 35, 40, 98]. We used the wrack width and height to capture variability across sites in an efficient way.

The variation in wrack composition in marine environments was estimated either by using visual quantification of material classes ‘as they look like’ [40, 99–102] or by sieving dried samples to obtain the particle size classes [96]. Here, we visually identified a high number of material classes (*k* = 20) with ascertained sources (e.g. lakeborn vs. landborn particles, anthropogenic wastes), followed by the estimation of percentage share per volume in situ.

The environmental factors that influence the abundance of wrack on marine coasts are diverse and complex. Many factors show a high degree of collinearity, which makes isolation of the primary important factors difficult, even with multivariate statistics (e.g. [32, 35, 102]). In our study design, we avoided a factorial design and instead assigned a cardinal scale level to all response and predictor variables. The main statistical analyses were performed using PLS regressions models unlike in previous studies.

A variety of results on tidal and non-tidal marine coasts and tidal freshwater estuaries, and the references herein, suggest that the following factor groups have an effect on wrack accumulation (Table 4):

1. conditions in offshore donor sites
2. conditions in upland donor sites, including tributaries
3. conditions of detachment, erosion, and transport
4. conditions in the recipient system
5. seasonality of environmental variables

**Table 4:** Factors influencing the amount of wrack in marine environments (literature) and in Lake Constance-Obersee (this study).

factor complex	factor	marine environment	Lake Constance-Obersee	comments
		references	predictor variables	
conditions in the offshore donor sites	spatial extension		width of the (vegetated) littoral platform (*WSUB*)	
	proximity to the recipient site	[37, 40]	upper macrophyte limit (*UDL10*, *UDL50*)	
	substrate type	[97]	sediment texture (*XG%*, *SUT%*)	
	phytomass productivity	[108]	not included in the model	approximately the same for the meadows of all *Chara* species
	growth form	[35]	not included in the model	approximately the same for all *Chara* species
	phenology, senescence	[31, 37, 40, 96]	not included in the model	approximately the same for all *Chara* species
conditions in upland donor sites	proximity to inflows	[34]	not relevant for 19 out of 20 sites	one site with a creek mouth nearby
	proximity to settlements		not included in the model	nearby settlements in all sites
conditions of detachment and transport	resistance of the plant organs against detach-ment and erosion	[109–111]	not included in the model	approximately the same for all *Chara* species
	wave forces	[28]	total effective fetch (*TEF*); total wind exposure (*TWE’3*); wind wave exposure (*WWE15*), ship wave exposure (*DCAT*)	
	buoyancy of the donor material	[112]	not included in the model	negative buoyancy for the stems of all *Chara* species and for other materials
conditions in the recipient system	tidal system	[31, 36]	not relevant	no diurnal water level changes in Lake Constance
	beach exposure	[31, 40, 113]	shoreline exposure (*ES*)	
	beach reflectivity	[37]	not included in the model	
	beach morphology (bays, horns)	[39, 114]	not relevant	no such rhythmic structures
	cross-shore profile, inclination	[35, 36, 40]	shore relief (*WEU*)	
	beach substratum and hydraulic roughness	[31, 97]	not included in the model	
seasonality	winds (velocity, direction)	[31, 36]	not included in the model	the same strong wind events for the lower wrack line of all sites
	wave forces	[31, 32]	not included in the model	depending on seasonality of winds (s. above)
	water level change	not relevant	not included in the model	identical for all sites

Unsurprisingly, the conditions for wrack formation in large inland lakes, e.g. Lake Constance-Obersee, differ from those on marine coasts. The crucial differences are (i) the lack of diurnal tides, (ii) the submerged vegetation type in the donor sites, (iii) the generally smaller dimensions of wind velocities, wave forces, fetch, and beach width, and (iv) the productivity and phenology (senescence) of the submerged vegetation in the donor system. However, there are also similarities in conditions between both environment types, e.g., the physical forces involved in suspension and transporting the sea-/lakeborne material, the morphology, inclination and roughness of the beach face, the seasonality of frequent strong wind events and the high availability of senescent plant material in the winter half-year. A special feature of Lake Constance (and a few other large Alpine lakes) is its regular water level cycle: a high water phase in June-July and a low water phase in February-March. We considered these predictable fluctuations when deciding upon predictor variables (Table 4).

During winter, the declining water level and high wind activity combined with the winterly die-off of macrophytes lead to the formation of wrack lines in Lake Constance-Obersee. These are deposited successively, so that the first wrack line formed lies highest up the beach. In this study, we focused on the youngest (lowermost) wrack line. Its formation could be traced back to three distinct formation events in 2019 and one event in 2020, respectively. The older wrack lines were caused by different events that could not be easily reconstructed in retrospect. However, the differences between the formation events of the two study years had no significant effects on the position, shape, volume and composition of the wrack lines. This suggests that site-specific conditions may be more important than hydrological and wind variability between years (Table 3).

### Width of the sublittoral zone

Shape and volume of the lower wrack line were most influenced by the width of the sublittoral zone (Table 3). The wider the sublittoral zone, the wider and higher the lower wrack line and thus the greater its volume (Figs 9 and 10). The sublittoral is where the *Chara* meadows grow, the remains of which make up most of the wrack material. The sublittoral zone is also largely congruent with the surf platform where deep-water waves are transformed (e.g. wave shoaling) and dissipated by friction at the lake bed and breaking [103]. Thereby, the wave energy interacts with the submerged macrophytes (e.g. breaking and tearing off senescent *Chara* stems), as well as with sediments or mollusc shells (e.g. sediment/shell resuspension and transport [104]). In the surf and wash zone, shortly before and during wave breaking, the wave orbitals are no longer circular and thus non-linear effects (e.g. Stokes drift) dominate, which drives shoreward transport of floating material and suspended particles getting deposited above the water line. With increasing width of the surf platform, the transport potential increases as wave energy dissipation takes place over longer distances and non-linear effects are more pronounced. Thus, the source zone of material that forms the wrack increases with the width of the surf platform.

### Width of the lower eulittoral zone

The width of the lower eulittoral zone had a similar, but smaller effect (Table 3) on wrack composition and shape. We assume that the width of the eulittoral zone influences swash processes (uprush–onshore flow, and backwash–offshore flow): in marine environments, greater swash generally occurs on flatter beaches [105]. Here, more sediments get transported by the uprush, resulting in net onshore sediment transport. On steeper beach faces, the sediment transport is dominated by the backwash [106, 107]. It is thus likely that gently sloped beaches also favour the stranding of suspended plant material at the end of the uprush zone, where the current velocity is zero. In accordance with this, Barreiro et al [32] found on tidal marine coasts a correlation of wrack biomass with the reciprocal value of the slope in the intertidal beach zone, and Reimer et al. [37] observed a relationship of wrack abundance with the Iribarren parameter of the near-shore surf zone. In both cases, high wave energy dissipation resulted in a high abundance of wrack material.

The position of the wrack line along the cross-shore profile (*ZWL*_*base*_) depended on the widths of the sublittoral and the eulittoral zones (Table 3): the narrower these zones were, the higher up the beach the wrack was washed (Fig 11). Generally, on a steep shore, most of the wave energy is dissipated in a narrow breaker zone near the water line, resulting in a stronger uprush which drives the wrack further up the beach.

### Deep water wave forces

The indicators of deep-water wave forces that significantly contributed to our models were total effective fetch, distance to the catamaran route and total wind exposure. A higher total effective fetch and a lower distance to the catamaran route (i.e. a higher deep-water wave energy input in both cases) resulted in a greater width and volume of the lowermost wrack line (Figs 9 and 10).

In general, the higher the wave energy entering the nearshore zone, the higher the shear stress in the water column, specifically on plant organs. In narrow and mostly steep sublittoral zones, most of the wave energy is dissipated in a narrow band. Since fewer macrophyte plants are present in these zones, those present are more likely to be uprooted or broken by wave shear. In contrast, in wide and more gently sloping sublittoral zones, macrophytes have a wider environment to grow and build up a high biomass. Moreover, waves entering these wide sublittoral zones dissipate their energy over a wider spatial range. The probability is high that there is a band somewhere in the sublittoral zone where a high critical shear stress meets a high biomass. Hence, under similar wind exposure and wave field conditions, sites with a wider sublittoral zone have a higher potential to form wrack lines composed of macrophytes and other substrates (Figs 9 and 10)

The other indicator of deep-water wave energy input, *WWE15*, which had a spatially higher resolution, played an insignificant role in the optimised PLS regression models, and was less informative than the more plausible quantities *TEF* and *DCAT*.

### Sediment texture

The sediment texture played only a minor role in this study. For example, the vertical position of the lowest wrack line was influenced by the percentage of coarse sediments (Table 3 and Fig 11). However, the combined effects of sediment properties and beach slope on energy dissipation of onshore traveling waves are very complex [115]. In general, wave energy dissipation rates are higher over immobile, rough bed forms than over mobile sandy lake beds or mud-flats [115]. In line with this Gilson et al. [97] found a generally lower wrack biomass on sandy sites than on pebble shores of marine tidal coasts in NW Ireland.

### Composition of the wrack material

At Lake Constance, the underwater vegetation is dominated by *Chara* species, with some *Potamogeton/Stuckenia* species. Remains from both taxa made up *M* = 34% of the lower wrack line. Similarly, on marine beaches the composition of wrack depends mainly on the wrack subsidies in the donor ecosystem [31, 96]. However, the diversity of wrack materials was higher than in marine environments (S4.3 Table in S1 Results), as mollusc remains and admixtures of terrestrial material were also abundant (Fig 7).

The percentages of anthropogenic wastes were low, even in the vicinity of settlements, with an average of 0.4%. This shows that Lake Constance shores are contaminated with anthropogenic litter as little as it is the case on urban shores in other lakes [33, 116–118]. Lazcano et al. [45] found a co-distribution of litter and particulate organic matter such as leaves and algae on Chicago beaches, Lake Michigan. Wrack may support the entrapment of anthropogenic litter, especially in the vicinity of artificial breakwaters [98]. Similarly, in the brackish semi-enclosed Baltic Sea, which has mainly wind-driven tides, the litter distribution on the beaches is related to the presence of beach wrack lines [119]: about 70% of litter items was found with wrack lines and 30% on or within the bare substrate. Menicagli et al. [34] found that the beach litter density was positively correlated to the proximity of major harbors on Mediterranean coasts while its composition was related to the proximity to both major harbors and rivers. However, our observations on Lake Constance-Obersee did not support these relationships.

In the marine environment, materials get diversely transported, depending on the physical properties of the source material, such as buoyancy [32, 112]. All materials in our study, with the exception of driftwood and some plastics, had a negative buoyancy. This included submerged plant material, e.g. *Chara* species due to carbonate incrustations on leaves and stems. Thus, we assume uniform transport conditions in Lake Constance-Obersee induced by waves and currents as described above. Little is known about other factors that determine the composition of marine wrack lines: Orr et al. [31] highlight shore exposure, Barreiro et al. [32] and Gómez et al. [35] emphasise seasonal influences. The latter factor was not considered in our survey but ought to be investigated in future studies.

High and constantly occurring deep water wave energy inputs due to the hourly catamaran passages all year round may favour the breaking and/or uprooting of *Chara* stems and the resuspension of mollusc shells (Fig 12). A gently sloped eulittoral zone favours onshore flow compared with backwash, thereby supporting the stranding of source materials as discussed above.

The proportion of terrestrial components (*MC31*, *MC33*) in the wrack increased significantly with increasing percentage of fine sediments in the beach surface (Table 2). This may be because fine-grained substrates favour the growth of riparian woody plants and *Phragmites* reeds over dwarf beach vegetation (Ostendorp, pers. obs.), thus increasing the availability of this kind of donor material.

Our studies on Lake Constance-Obersee, an oligotrophic Alpine lake with almost natural water level fluctuations, demonstrate that considerable alluvial deposits of submerged plant and mollusc remains can occur on poorly vegetated shores, similar to marine beaches. The conditions for wrack line formation differ from marine coasts, mainly due to the absence of diurnal tides, a lower wave load, and the variable persistence of the wrack. However, essentially the same hydrodynamic and relief factors occur, which are complexly correlated and act simultaneously. Our results are well explained by the general laws of interaction between fetch, wind and ship waves, breakers, up-rush and down-wash, and beach inclination, as best described for marine environments.

## Conclusions

The relevant predictors of wrack formation at Lakes Constance shores were extracted and their influence on the dimensions, the specific volume and the particulate composition of the wrack lines (response) was analysed using PLS regressions. We found that:

- the width of the lower eulittoral zone as an indicator of the swash conditions (up-rush vs. down-wash),
- high exposure to wind waves, as indicated by the total effective fetch, and high exposure to ship waves of the catamaran, and
- the width of the sublittoral zone as an indicator of (i) the availability of source material (*Chara* spp.) and (ii) the wave energy dissipation of the incoming deep-water waves

were the most important environmental factors in determining wrack formation and composition. In contrast, sediment texture played only a minor role. The significant influence of the catamaran ferry showed that anthropogenic factors can enhance the occurrence and specificity of wrack lines. The width of the lower eulittoral zone and a high ship wave exposure were most influential on the composition of wrack lines, leading to high proportions of lake-borne components (*Chara* remains, mollusc shells), while the reverse was true for land-based components. Anthropogenic waste materials were only present in small proportions.

Our investigations only refer to the wrack lines that were formed at the end of the winter low-water period, and could be assigned to distinct causative strong wind events. Occasional observations at Lake Constance demonstrate that during the period of declining water levels (Aug to Jan), mostly small wrack lines are subsequently formed. They have different particulate compositions (Ostendorp, pers. observations) and are presumably subject to different formation conditions.

The persistence and the ecological importance of wrack lines at Lake Constance and other inland lakes as well as the temporal variability, depending on hydrological and weather conditions, have not yet been sufficiently investigated. However, it is quite possible that they are an essential factor for the ‘favourable conservation status’ of dwarf eulittoral vegetation under Art. 1 (e) of the FFH Directive (e.g. FFH habitat types 3110 and 3130 [77]).

It ought to be noted that wrack lines are natural components of pristine shore ecosystems at Lake Constance. The ecological importance of wrack for nutrient balance and for food chains in the eulittoral zone of inland lakes has not yet been studied in detail. However, it can be assumed that lakeshore wrack plays a comparable role to marine coast wrack. As in several marine coastal areas [120], Lake Constance’s shores are cleaned up by water management agencies, local municipalities and volunteer groups. Wrack material, including coarse woody debris is removed, mainly for aesthetic reasons or with consideration for bathers and tourists, however the potential ecological impacts have remained unconsidered until now. Without a more precise knowledge of the function of wrack lines in the lakeshore ecosystem, these activities should therefore be carried out very restrictively.

## Supporting information

S1 Data Characteristics of the study sites, environmental variables (predictors), properties of wrack lines (response variables), composition of wrack lines (response variables). (PDF)

S1 Methods Calculation of total effective fetch (*TEF*) and wind exposure (*TWE*). (PDF)

S2 Methods Statistical evaluation–PLS regression. (PDF)

S1 Results Normalising transformations of the response and the predictor variables, normalising transformations of the response variables of the wrack line composition, composition of the lowermost wrack lines. (PDF)

S1 Annex (DOCX)
