# Supplementary material for: Wrack line formation and composition on shores of a large Alpine lake: The role of littoral topography and wave exposure
Source: PLoS One. 2023 Nov 30;18(11):e0294752. doi: 10.1371/journal.pone.0294752 (PMC10688906; doi:10.1371/journal.pone.0294752)
Supplement: S1 Data — (PDF) [file pone.0294752.s001.pdf]

Supporting Information

Wrack line formation and composition on shores of a large Alpine lake: the role of littoral topography and wave exposure

Wolfgang Ostendorp

ORCID: 0000-0002-2171-7356

Environmental Physics Group, Limnological Institute, University of Konstanz, Konstanz, Germany

Hilmar Hofmann

ORCID: 0000-0001-6140-5886

Staff Unit Sustainability, University of Konstanz, Konstanz, Germany

Jens Peter Armbruster

ORCID: 0000-0003-4137-7675

Institute for Landscape Ecology and Nature Conservation (ILN) Südwest, Kirchheim u.T., Germany

S1 – Data

Table S1.1 - Characteristics of the study sites.
Table S1.2 – Environmental variables (predictors)
Table S1.3 – Properties of wrack lines (response variables)
Table S1.4 – Composition of wrack lines (response variables)

Table S1.1: Characteristics of the study sites. *ES* – shoreline exposure in lakeward direction and perpendicular to the shoreline, *TEF* – effective fetch, length of the study site (length of uninterrupted, natural shore section), *WEU* – width of the shore between mean average water line (mMWL, 395.24 m NN) and mean low water line (mNWL, 395.58 m NN), *WSUB* – width of the shore between mean low water line (mNWL, 395.58 m) and shelf break (here assumed to be 390.50 m NN), slope of the eulittoral and the sublittoral zone.

**Shore section**	**Position**	**Survey years**	***ES***	***TEF***	**Length of the study site**	***WEU***	***WSUB***	**Inclination eulittoral**	**Inclintion sublittoral**
	UTM 32N (X, Y)		(° wind rose)	(km)	(m)	(m)	(m)	(°)	(°)
BODM	503724; 5293251	2020	56	59.7	n/a	11	67	3.4	4.1
LUDW	503508; 5295881	2019, 2020	195	90.0	150	35	90	1.1	4.2
UEBW	509711; 5291564	2019, 2020	238	57.9	120	10	109	3.7	2.3
UMMP	516488; 5287758	2019, 2020	218	79.0	25	20	149	1.9	1.8
UUHL	517454; 5284952	2019, 2020	235	108.3	100	22	162	1.7	1.6
UUHE	518472; 5283633	2019, 2020	226	150.1	200	9	53	4.1	5.2
HALT	522169; 5280869	2019, 2020	204	179.9	80	11	89	3.4	2.9
HADY	522688; 5280566	2019, 2020	210	191.0	250	14	121	2.7	2.1
HAGN	524745; 5279574	2019, 2020	220	191.0	120	23	205	1.6	1.3
KIPP	525909; 5278828	2019, 2020	213	246.0	50	15	203	2.5	1.2
IMSB	527037; 5278769	2019, 2020	174	217.7	15	13	258	2.9	0.9
IMMS	528078; 5279205	2019, 2020	184	202.9	40	16	483	2.3	0.5
FNFB	530641; 5279458	2019, 2020	218	222.6	34	82	820	0.5	0.3
FNSM	533825; 5278067	2019, 2020	207	224.0	60	43	454	0.9	0.6
FNSB	534112; 5277938	2019	220	223.8	50	35	456	1.1	0.5
FNSC	534471; 5277656	2019, 2020	185	223.6	85	14	427	2.7	0.6
KRTU	543559; 5270465	2019, 2020	184	143.9	175	5	77	7.4	3.2
NONN	545286; 5270249	2019, 2020	244	147.7	360	4	142	9.2	1.7
BOTT	516041; 5276698	2020	63	165.0	n/a	16	152	2.3	1.7
MUEN	517778; 5275534	2020	18	148.7	n/a	12	401	3.1	0.6

Table S1.2: Environmental variables (predictors). *XG%*, *SUT%* – pooled grain sized classes cobbles+gravel, and sand+silt+clay, *TWE'3* – total wind exposure based on wind data from the nearest weather station, only winds of ≥3 Bft, *WWE15* – exposure to wind waves exeeding significant wave height of 15 cm, *DCAT* – minimum distance of a shore section from the route of the catamaran in 2019 or 2020, *UDL10*, *UDL50* – level above/below long term average mean water (*MW*) at the landward limit of the submerged macrophyte vegetation when falling below a critical coverage of 10% respectively 50% in survey years 2019 and 2020.

**Shore section**	***XG%***	***SUT%***	***TWE'3***	***WWE15***	***DCAT (2019/20)***	***UDL10 (2019)***	***UDL10 (2020)***	***UDL50 (2019)***	***UDL50 (2020)***
	(%)	(%)	(m)	(%)	(km)	(m)	(m)	(m)	(m)
BODM	40	20	1673	0.1	17.61		-0.25		-0.38
LUDW	0	20	486	0.2	19.96	0.03	0.03	0.03	0.03
UEBW	20	70	1304	5.5	13.51	-0.18	-0.25	-0.18	-0.27
UMMP	20	40	2499	6.2	9.55	-0.14	-0.14	-0.14	-0.14
UUHL	5	70	1963	5.6	7.02	-0.32	-0.32	-0.53	-0.53
UUHE	60	10	2314	6.4	5.83	-1.07	-1.07	-1.07	-1.07
HALT	50	10	3842	15.3	3.44	-0.48	-0.48	-0.62	-0.62
HADY	60	5	4096	15.9	3.20	-0.41	-0.41	-0.55	-0.55
HAGN	40	10	4671	16.1	2.36	-0.34	-0.45	-0.55	-0.71
KIPP	50	10	15234	20.4	1.71	-0.58	-0.58	-0.68	-0.68
IMSB	90	5	9232	19.4	1.74	-0.10	-0.01	-0.52	-0.31
IMMS	5	35	9673	17.7	2.27	-0.08	-0.08	-0.08	-0.08
FNFB	0	95	14486	18.1	2.75	-0.19	-0.19	-0.19	-0.19
FNSM	0	60	18847	17.8	1.82	-0.61	-0.29	-0.53	-0.63
FNSB	15	25	18910	17.0	1.71	-0.19		-0.19	
FNSC	60	20	19271	19.9	1.45	-0.37	-0.37	-0.37	-0.37
KRTU	20	40	12157	29.9	9.78	-1.56	-1.56	-1.87	-1.87
NONN	98	0	15908	22.8	11.27	-1.01	-1.01	-2.21	-2.21
BOTT	5	75	898	4.5	1.33		-0.38		-0.85
MUEN	20	60	1015	3.0	2.30		-0.45		-0.54

Table S1.3: Properties of wrack lines. *NWL* – number of wrack lines, *HWL* – average thickness of wrack near to the crest of the lowermost wrack line, *WWL* – average width of the lowermost wrack line, *VWL* – specific volume of the lowermost wrack line, *Vtotal* – specific volume of all wrack lines, *ZWLbase* – level of the base of the lowermost wrack line under its crest (above/below long-term average mean water level, *MW*), no data for 2019.

**Shore section**	***NWL* *(2019)***	***NWL* *(2020)***	***HWL* *(2019)***	***HWL* *(2020)***	***WWL* *(2019)***	***WWL* *(2020)***	***VWL* *(2019)***	***VWL* *(2020)***	***Vtotal* *(2019)***	***Vtotal* *(2020)***	***ZWLbase* *(2020)***
	(-)	(-)	(m)	(m)	(m)	(m)	(m^3/m)	(m^3/m)	(m^3/m)	(m^3/m)	(m)
BODM		1		0.06		0.38		0.011		0.011	
LUDW	3	1	0.05	0.05	1.00	0.10	0.025	0.002	0.063	0.002	0.07
UEBW	1	1	0.06	0.13	0.50	0.90	0.015	0.057	0.015	0.057	
UMMP	1	2	0.15	0.18	1.50	0.93	0.113	0.086	0.113	0.142	0.07
UUHL	1	1	0.07	0.14	2.00	1.11	0.070	0.077	0.070	0.077	
UUHE	1	1	0.04	0.09	0.70	0.46	0.014	0.020	0.014	0.020	0.27
HALT	1	1	0.07	0.03	1.00	0.79	0.035	0.011	0.035	0.011	0.35
HADY	1	1	0.05	0.07	1.50	0.98	0.038	0.033	0.038	0.033	0.36
HAGN	1	1	0.09	0.05	2.00	2.93	0.090	0.073	0.090	0.073	0.27
KIPP	1	2	0.03	0.15	2.00	1.48	0.030	0.111	0.030	0.475	0.17
IMSB	1	2	0.29	0.11	3.00	2.17	0.435	0.119	0.435	0.124	0.25
IMMS	1	1	0.17	0.15	3.00	2.29	0.255	0.166	0.255	0.166	-0.05
FNFB	1	2	0.14	0.18	2.00	3.82	0.140	0.347	0.140	0.401	0.04
FNSM	1	2	0.31	0.45	2.50	3.15	0.388	0.705	0.388	0.729	-0.12
FNSB	2		0.7		3.50		1.225		1.475		
FNSC	1	1	0.03	0.13	0.70	0.49	0.011	0.032	0.011	0.032	0.02
KRTU	1	3	0.08	0.04	0.75	0.13	0.030	0.003	0.030	0.157	0.02
NONN	2	2	0.1	0.30	0.50	2.10	0.025	0.315	0.027	0.375	0.33
BOTT		1		0.28		4.20		0.581		0.581	0.27
MUEN		1		0.07		0.48		0.016		0.016	0.07

Table S1.4: Composition of the lowermost wrack line. Main components were: *MC11* – charophyte algae remains, *MC22* – gravel, *MC23* – mollusc shells, *MC31* – dead foliage from reparian trees, *MC32* – branch material, *MC33* – reed (*Phragmites australis*) stems and leaves.

**Shore section**	***MC11* (2019)**	***MC11* (2020)**	***MC22* (2020)**	***MC22* (2020)**	***MC23* (2019)**	***MC23* (2020)**	***MC31* (2019)**	***MC31* (2020)**	***MC32* (2019)**	***MC32* (2020)**	***MC33* (2019)**	***MC33* (2020)**
	(%)	(%)	(%)	(%)	(%)	(%)	(%)	(%)	(%)	(%)	(%)	(%)
BODM		0.1		0.0		2.0		60.0		31.8		3.0
LUDW	60.0	13.0	0.0	74.9	25.0	1.0	5.0	0.1	5.0	0.9	5.0	10.0
UEBW	0.0	3.0	0.0	3.0	60.0	25.0	5.0	3.0	10.0	1.0	25.0	56.2
UMMP	30.0	28.0	0.0	0.0	60.0	64.4	2.0	0.1	2.8	5.0	5.0	1.0
UUHL	20.0	55.0	0.0	12.0	56.0	5.0	5.0	2.0	2.0	1.0	2.0	0.8
UUHE	20.0	73.8	0.0	0.0	71.0	11.0	3.0	3.0	3.0	8.0	3.0	1.0
HALT	0.5	6.0	0.0	1.0	96.3	86.3	1.0	0.0	1.0	1.0	0.0	0.5
HADY	7.0	15.0	0.0	0.0	43.0	45.0	3.0	1.0	4.0	3.0	0.5	0.0
HAGN	29.4	4.0	2.0	0.0	64.0	94.6	1.0	0.1	1.0	0.1	0.5	0.1
KIPP	4.0	5.0	23.9	15.0	45.0	66.6	1.0	0.5	2.0	1.0	8.0	0.5
IMSB	10.0	83.2	0.0	0.0	79.8	0.5	1.0	5.0	1.0	11.0	0.0	0.1
IMMS	27.3	84.7	0.0	0.0	70.1	10.0	0.5	1.0	1.0	3.0	0.5	1.0
FNFB	44.0	60.0	0.0	0.0	10.0	15.0	30.0	1.0	3.0	5.0	1.0	11.0
FNSM	88.3	95.0	2.0	0.0	5.0	5.0	1.0	0.0	0.5	0.0	0.1	0.0
FNSB	91.0		0.0		3.0		0.2		0.1		0.0	
FNSC	80.4	98.8	0.0	0.0	15.0	1.0	2.0	0.1	1.0	0.1	1.0	0.0
KRTU	2.0	0.0	0.0	0.0	0.1	0.0	25.0	18.0	71.3	29.0	0.0	51.9
NONN	0.0	0.0	0.0	98.5	0.0	0.1	5.0	0.1	90.0	1.0	0.0	0.0
BOTT		0.0		1.0		5.0		79.3		0.1		0.1
MUEN		0.0		0.0		5.0		7.0		30.0		29.9
