# Supplementary material for: Wrack line formation and composition on shores of a large Alpine lake: The role of littoral topography and wave exposure
Source: PLoS One. 2023 Nov 30;18(11):e0294752. doi: 10.1371/journal.pone.0294752 (PMC10688906; doi:10.1371/journal.pone.0294752)
Supplement: S1 Methods — (PDF) [file pone.0294752.s002.pdf]

Supporting Information

Wrack line formation and composition on shores of a large Alpine lake: the role of littoral topography and wave exposure

Wolfgang Ostendorp

ORCID: 0000-0002-2171-7356

Environmental Physics Group, Limnological Institute, University of Konstanz, Konstanz, Germany

Hilmar Hofmann

ORCID: 0000-0001-6140-5886

Staff Unit Sustainability, University of Konstanz, Konstanz, Germany

Jens Peter Armbruster

ORCID: 0000-0003-4137-7675

Institute for Landscape Ecology and Nature Conservation (ILN) Südwest, Kirchheim u.T., Germany

S2 – Methods:

Calculation of total effective fetch (*TEF*) and wind exposure (*TWE*)

S2. Methods: Calculation of total effective fetch (*TEF*) and wind exposure (*TWE*)

S2.1 Total effective Fetch (*TEF*)

The calculation of the effective fetch *EF* /m is based on the single beam fetch $F_{sb}(\alpha)$ which is the free wind path over the lake surface between the points P_0 and $P_E(\alpha)$. P_0 is the point of interest for which the fetch is calculated; this point lies by definition on the 4 m depth contour line (391 m NHN) as close as possible to the study site. $P_E(\alpha)$ is the end point, i.e. the intersection of a beam with a directional angle α of P_0 with the z = 4 m depth contour line on the opposite side of the lake. The 4 m depth contour corresponds to the wave base (0.5 L, L /m – characteristic wind wave length, usually $6 < L < 11$ m in Lake Constance Obersee), above which ground contact and wave energy dissipation occurs during heavy swell (Hofmann et al. 2008). For simplicity, it was assumed that in the littoral area with $z < 4$ m, no waves were generated during offshore winds due to elevation of the horizon (horizontal shading), and that during onshore winds, the incoming waves lost energy compared to the deep water waves due to bottom friction.

The single beam fetch $F_{sb}(\alpha)$ was calculated for the directional angles α = 10° - 360° with 10° intervals. If the beam, starting from P_0 on the 4 m bathymetric line, extended over littoral areas (z < 4 m), this straight line segment was set to 0 m.

The effective fetch $EF(\alpha)$ of the direction angle α considers not only the main direction in view (i.e. the single beam fetch), but also the adjacent beams $F_{sb}(\alpha\text{-}10°)$ and $F_{sb}(\alpha\text{+}10°)$ (Keddy 1982, Rohweder et al. 2012). The effective fetch $EF(\alpha)$ is then calculated as the arithmetic mean of these three values (Eq 1).

$$EF(\alpha) = \frac{1}{3} \times (F_{sb}(\alpha) + F_{sb}(\alpha - 10°) + F_{sb}(\alpha + 10°)) \qquad \text{Eq. (1)}$$

The total effective fetch *TEF* /m is the sum of all angle-related effective fetch lengths over the entire 36-part compass rose (Eq 2):

$$TEF = \sum_{n(\alpha)=1}^{36} EF\,(\alpha) \qquad \text{Eq. (1)}$$

S2.2 Wind exposure (*TWE*)

The total wind exposure (*TWE*) is defined here as the effective fetch of a point on the 4 m bathymetric line weighted by the relative duration and forces of winds' overall direction angles.

The wind data were based on hourly mean values (speed v /m s^{-1}, direction angle α /°) from seven meteorological stations on the northern and southern shore of Lake Constance, operated by the German Meteorological Service (Deutscher Wetterdienst, DWD Offenbach) and MeteoSwiss (Federal Office of Meteorology and Climatology, Berne). The data from 01 Oct to 31 Mar of the years 2018/19 and 2019/20 were evaluated.

S2.2.1 Considering all wind strengths classes

The relative frequency f of a wind force class k for a direction angle α, $f_k(\alpha)$ is the number of hourly readings in that class divided by the total number of hourly readings recorded for all wind force classes and direction angle classes in a predefined period (here 01 Oct to 31 Mar in the years 2018/19).

The wind force classes k /Bf were defined using the 12-part Beaufort wind force scale including class 0 (calm). The directional angle classes i had a width of 10° and ran from 6-15° to 356-5°. The angle α is the bisectrix of the class in view. The overall sum of all $f_{k,i}$ is 1 (Eq 3).

$$1 = \sum_{i=1}^{36} \sum_{k=0}^{12} f_{k,i} \qquad \text{Eq. (3)}$$

The effective fetch *EF* weighted by wind speed and relative wind frequency of a given direction angle α, $EFw(\alpha)$ /m is calculated as

$$EFw(\alpha) = \frac{1}{v_0} \sum_{k=0}^{12} v_k(\alpha) \times f_k(\alpha) \times EF(\alpha) \qquad \text{Eq. (4)}$$

where v is the mean wind velocity (m s^{-1}) recalculated from the Beaufort wind force class k using the empirical relationship v = 0.836 m $s^{-1} \times k^{2/3}$ (BEER 1997). v_0 is 1 /m s^{-1} so that *EFw* has the same dimension as *EF*. The wind exposure (*TWE* /m) at P_0 was calculated as the sum of weighted effective fetch values across all direction angles, defined as

$$TWE = \sum_{i=1}^{36} EFw \qquad \text{Eq. (5)}$$

where index i represents the directional angle classes. All wind force classes were used for the calculations.

To calculate *TWE* at point P_0, the wind data of the weather station Konstanz were used, which was found to represent best the wind forces and wind directions in the western part of Lake Constance.

Alternatively, the weather station next to P_0 was used (*TWE'*). Furthermore, the weighted wind exposure *TWE''* was calculated for each point P_0, whereby the values of the m = 7 weather stations were weighted with $1/d^2$ (d /m – distance between P_0 and the weather station), defined as

$$TWE'' = \frac{\sum_{m=1}^{7}(E_{wind,m} \times d_m^{-2})}{\sum_{m=1}^{7} d_m^{-2}} \qquad \text{Eq. (2)}$$

S2.2.2 Considering wind strength classes above a threshold value

All wind force classes ($k \geq 0$ Bf) were used for the calculations, as described above. We additionally defined wind classes by setting threshold wind force values (KEDDY 1982) of 3 Bft (gentle breeze and stronger, *TWE3*, *TWE'3*, *TWE''3*) and 5 Bft (fresh breeze and stronger, *TWE'5*, *TWE''5*).

References:

BEER, T. 1997. Environmental Oceanography. CRC Press. ISBN 0-8493-8425-7.

HOFMANN, H.; LORKE, A. and PEETERS, F. (2008). The relative importance of wind and ship waves in the littoral zone of a large lake. – Limnology and Oceanography 53(1): 368-380.

KEDDY, P. A. 1982. Quantifying within-lake gradients of wave energy: interrelationships of wave energy, substrate particle size and shoreline plants in Axe Lake, Ontario. – Aquatic Botany 14: 41-58.

ROHWEDER, J.; ROGALA, J. T., JOHNSON, B. L., ANDERSON, D., CLARK, S., CHAMBERLIN, F., Potter, D. and RUNYON, K. 2012. Application of wind fetch and wave models for habitat rehabilitation and enhancement projects – 2012 Update. Contract report prepared for U.S. Army Corps of Engineers' Upper Mississippi River Restoration. – Environmental Management Program: 52.
