# Supplementary material for: Wrack line formation and composition on shores of a large Alpine lake: The role of littoral topography and wave exposure
Source: PLoS One. 2023 Nov 30;18(11):e0294752. doi: 10.1371/journal.pone.0294752 (PMC10688906; doi:10.1371/journal.pone.0294752)
Supplement: S2 Methods — (PDF) [file pone.0294752.s003.pdf]

Supporting Information

Wrack line formation and composition on shores of a large Alpine lake: the role of littoral topography and wave exposure

Wolfgang Ostendorp

ORCID: 0000-0002-2171-7356

Environmental Physics Group, Limnological Institute, University of Konstanz, Konstanz, Germany

Hilmar Hofmann

ORCID: 0000-0001-6140-5886

Staff Unit Sustainability, University of Konstanz, Konstanz, Germany

Jens Peter Armbruster

ORCID: 0000-0003-4137-7675

Institute for Landscape Ecology and Nature Conservation (ILN) Südwest, Kirchheim u.T., Germany

S3 – Methods:

Statistical evaluation – PLS regression

The data of the predictor and response variables were tested for normal distribution (Shapiro-Wilk test) and, if necessary, subjected to a normalising transformation (mostly Johnson S_B or S_U transformation). The transformed variables were tested for collinearity (correlation matrix, Pearson correlation coefficient, Kendall's τ, partial correlation coefficients).

The influence of environmental variables was analysed using partial least squares regression (PLS). The method has favourable properties for the analysis of ecological data sets with

- both cardinal and categorical variables,
- a relatively small number of observations,
- a large number of highly correlated predictor variables (Xs, environmental variable),
- a non-normal distribution of many predictors (Xs), and
- a relatively small signal to noise ratio in the relationship between predictors and responses
- multivariate datasets (simultaneous effects on >1 correlated response variable)

(COX & GAUDARD 2013). The PLS regression was carried out with JMP® 14.

The first part of the PLS regression served to identify the model-relevant predictor variables from the collective of 15 variables. For this purpose, we first used the NIPALS (Nonlinear Iterative Partial Least Squares) algorithm (without validation) with a predefined number of two factors (i.e. principal components, projection axes). The algorithm produced the explained variance of the respective response variables, the cumulative variance of the predictor variables read in and the number of predictor variables that exceeded the threshold *VIP* (Variable Importance for the Projection), here: *VIP* = 0.8 or 0.9 as recommended by WOLD et al. 1995, p. 213 and WOLD et al. 2001, p. 123). The following programme run was carried out with these successful variables. After about three to six runs, in which only the relevant variables with VIP > 0.8 (or > 0.9) were kept for the successive run, the explained Y and cumulative X variances and a constant set of four predictors at maximum were obtained.

This pruned model was used in the second part of the PLS regression to determine the optimal number of factors after cross-validation with the leave-one-out method. The optimal number of factors was assumed to be the number for which the root of the PRESS statistic (Predicted Residual Sum of Squares) reached a minimum. Furthermore, the maximum of Q2 and the increase in the R2Y value were used as criteria. Almost all regressions resulted in only one latent factor onto which all predictors were projected.

In some cases where the first part of the PLS regression did not show stable convergence, zero was determined as the optimal factor number. This meant that there was no meaningful dependence on the predictors, and the response variable in view was best described by its mean value. This was tested by calculating a model with one factor (without cross-validation) and checking the goodness of fit of the model (R^2, p>|t|) by comparison with the measured data. In all these cases, the correlation coefficient was not significantly different from zero.

The goodness of fit of the model was assessed by measuring the correlation between the predicted and measured response variables (R^2, p>|t|). The low sample size prevented separation of the data into training and test sets. Only significant ($p < 0.05$) models were discussed. Multivariate outliers were identified using the Hotelling's T^2 plot with an upper control limit UCL ($p = 0.05$). In most cases, no multivariate outliers occurred. Any outlier that occurred were excluded and the calculation was repeated until all outliers were removed. We noted that the identification of relevant predictors in the first step of the PLS regression was not very stable against outliers.

Additionally, the multivariate residuals were graphically tested for deviations from the normal distribution (normal-quantile plot), as well as for independence (plot of the residuals over the predicted response variable). All data analyses indicated that the distribution was approximately normal and that samples were independent of each other.

The relative importance of a predictor included in the model was estimated using the *VIP* value, the loading on the factor(s) and the sign and absolute value of $\hat{b}$. $\hat{b}$ is the coefficient of the standardised (= centred and scaled) predictor variable in the pruned multivariate model. A high *VIP* value and a high loading indicate a good correlation of the variables with the factor and a correspondingly high relevance for the quality of the model. Variables with a high absolute value of $\hat{b}$ exert a stronger influence on the response variable than those

with a lower absolute value. The direction of the influence, amplifying or weakening, is given by the sign of $\hat{b}$.

References:

COX, I. and GAUDARD, M. 2013. Discovering Partial Least Squares with JMP®. 308 S., SAS Institute, Inc., Cary, North Carolina.

WOLD, S. 1995. PLS for Multivariate Linear Modeling. In: VAN DE WATERBEEMD, H. (ed.): Chemometric Methods in Molecular Design. Methods and Principles in Medicinal Chemistry, New York: VCH.

WOLD, S.; SJÖSTRÖM, M. and ERIKSSON, L. 2001. PLS-Regression: A Basic Tool of Chemometrics – Chemometrics and Intelligent Laboratory Systems, 58:2, 109-130.
