# Supplementary material for: Wrack line formation and composition on shores of a large Alpine lake: The role of littoral topography and wave exposure
Source: PLoS One. 2023 Nov 30;18(11):e0294752. doi: 10.1371/journal.pone.0294752 (PMC10688906; doi:10.1371/journal.pone.0294752)
Supplement: S1 Results — (PDF) [file pone.0294752.s004.pdf]

Supporting Information

Wrack line formation and composition on shores of a large Alpine lake: the role of littoral topography and wave exposure

Wolfgang Ostendorp

ORCID: 0000-0002-2171-7356

Environmental Physics Group, Limnological Institute, University of Konstanz, Konstanz, Germany

Hilmar Hofmann

ORCID: 0000-0001-6140-5886

Staff Unit Sustainability, University of Konstanz, Konstanz, Germany

Jens Peter Armbruster

ORCID: 0000-0003-4137-7675

Institute for Landscape Ecology and Nature Conservation (ILN) Südwest, Kirchheim u.T., Germany

S4 – Results.

Table S4.1 – Normalising transformations of the response and the predictor variables

Table S4.2 – Normalising transformations of the response variables of the wrack line composition

Table S4.3 – Composition of the lowermost wrack lines

Table S4.1: Normalising transformations of the response and the predictor variables. Results of the Shapiro-Wilk test, $p<|W|$. Transformations:

Johnson S_U : $Y^* = \text{arcsinh}((Y - a)/ b) \times c + d$
Johnson S_B : $Y^* = \log_e((Y - a) / (b - Y)) \times c + d$

See Annex for symbols. All response variables refer to the lowermost wrack line, except *NWL* and *Vtotal* which refer to all wrack line at a site. m±MW – above or below average mean water.

**variable**	**trans-formation**	**coefficients**				**sample size**	**normality test**
		**a**	**b**	**c**	**d**	**n**	$p<|W|$
Zveg /m ± MW	Johnson S_U	-0.28003	0.42147	1.3986	-1.0908	26	0.264
NWL / -	none					36	<0.0001
WWL1 /m	Johnson S_B	-0.25530	5.4485	0.86932	0.96788	36	0.763
HWL1 /m	Johnson S_U	0.02103	0.00351	0.93400	-3.4547	36	0.858
VWL1 /m^2	Johnson S_U	0.00022	0.00365	0.69079	-2.4125	36	0.804
Vtotal /m^2	Johnson S_B	-0.00590	5.2088	0.75883	3.0864	36	0.342
$ZWL1_{top}$ /m ± MW	Johnson S_B	0.00453	0.99303	0.93386	0.64174	16	0.807
$ZWL1_{base}$ / m ± MW	Johnson S_B	-0.17735	0.36250	0.58036	-0.43828	16	0.450
WWE15 /%	none					20	0.102
TWE'3Bft /m	Johnson S_B	459.77	1951.9	0.32996	0.20132	20	0.852
DCAT /km	Johnson S_B	1.1985	21.453	0.39584	0.66488	20	0.528
ES /°	Johnson S_U	252.70	0.000074	1.3590	18.974	20	0.497
TEF /m	none					20	0.124
WEU /m	Johnson S_U	9.4978	4.4238	0.85567	-0.91494	20	0.962
WSUB /m	Johnson S_U	33.026	0.00411	0.90445	-9.8729	20	0.578
UDL10 /m±MW	Johnson S_U	0.16589	0.000128	1.5941	14.279	20	0.548
OMG50 / m±MW	Johnson S_U	0.18506	0.000651	1.4969	11.353	20	0.240
XG% /%	Johnson S_B	-44.721	101.60	0.76789	-1.2086	20	0.644
SUT% /%	Johnson S_B	-1.6456	140.56	0.76336	1.1567	20	0.695

Table S4.2: Normalising transformations of the response variables of the wrack line composition. Results of the Shapiro-Wilk test, *p*<|*W*|. Transformations:

Johnson S_U : $Y^* = \text{arcsinh}((Y - a)/ b) \times c + d$
Johnson S_B : $Y^* = \log_e((Y - a) / (b - Y)) \times c + d$

See Table S4.3 for symbols. All response variables refer to all wrack lines at a site. Normality test – Shapiro Wilk Test.

**variable**	**trans-formation**	**coefficients**				**sample size**	**normality test**
		**a**	**b**	**c**	**d**	**n**	***p*<|WSW|**
MC11 /%	Johnson S_B	-0.03417	99.131	0.27688	0.73577	46	0.006
MC22 /%	no transf.					46	<0.001
MC23 /%	Johnson S_B	-0.000415	96.593	0.30511	0.61508	46	0.172
MC31 /%	Johnson S_U	-0.04912	0.36212	0.63932	-1.5047	46	0.034
MC32 /%	Johnson S_B	-0.01753	235.92	0.49691	2.1676	46	0.234
MC33 /%	Johnson S_B	-0.001529	58.250	0.26780	1.4042	46	0.003
MC1 /%	Johnson S_B	-0.12151	98.914	0.34192	0.54343	46	0.243
MC2 /%	Johnson S_B	-0.65123	99.019	0.41108	0.21613	46	0.509
MC3 /%	Johnson S_B	-0.10963	314.87	0.59084	2.0164	46	0.234
MC4 /%	Johnson S_B	-0.000794	6.0151	0.33751	1.5909	46	<0.0001

Table S4.3: Composition of the lowermost wrack lines (%). freq – no. of sites where the class of material was present (max. 36 sites, years 2019 and 2020 pooled). *Md*, Q_{25}, Q_{75} – median, 25% and 75% percentiles. Material classes MC35 and MC44 were only found in higher wrack lines.

material class	description	freq	min	max	*Md*	Q_{25}	Q_{75}
		-	%	%	%	%	%
MC11	remains of *Chara* spp	30	0.0	98.8	17.5	2.8	60.0
MC12	remains of *Potamogeton*/*Stuckenia*	15	0.0	35.0	0.0	0.0	1.0
MC13	remains of *Elodea canadensis*	1	0.0	5.0	0.0	0.0	0.0
MC14	remains of *Myriophyllum spicatum*	1	0.0	0.1	0.0	0.0	0.0
MC15	*Fontinalis antipyretica* clumps	1	0.0	0.9	0.0	0.0	0.0
MC16	filamentous green algae	1	0.0	8.0	0.0	0.0	0.0
MC21	sand	12	0.0	24.0	0.0	0.0	3.7
MC22	gravel	11	0.0	98.5	0.0	0.0	1.0
MC23	mollusc shells and fragments	35	0.0	96.3	15.0	4.5	61.0
MC31	foliage	35	0.0	79.3	1.5	0.5	5.0
MC32	wood branch fragments	36	0.0	90.0	2.0	1.0	5.0
MC33	*Phragmites australis* culm fragments and leaves	29	0.0	56.2	0.7	0.1	3.5
MC34	terrestrial fruits and seed	27	0.0	29.5	0.1	0.0	0.5
MC35	*Viscum album* foliage	0	0.0	0.0	0.0	0.0	0.0
MC41	nrick rubble	12	0.0	3.0	0.0	0.0	0.1
MC42	glass fragments	7	0.0	1.0	0.0	0.0	0.0
MC43	aluminium (tin lids etc.)	2	0.0	0.1	0.0	0.0	0.0
MC44	iron parts	0	0.0	0.0	0.0	0.0	0.0
MC45	macro plastics	18	0.0	5.0	0.1	0.0	0.1
MC46	charcoal	1	0.0	0.0	0.0	0.0	0.0
