# Supplementary material for: Wrack line formation and composition on shores of a large Alpine lake: The role of littoral topography and wave exposure
Source: PLoS One. 2023 Nov 30;18(11):e0294752. doi: 10.1371/journal.pone.0294752 (PMC10688906; doi:10.1371/journal.pone.0294752)
Supplement: S1 Annex — (DOCX) [file pone.0294752.s005.docx]

Annex

**Abbreviations and Symbols**

| **Symbol** | **Unit** | **Description** |
| --- | --- | --- |
| *b* |  | coefficient of a predictor variable in the bivariate or multiple regression model or in the PLS regression model |
| $\hat{b}$ | - | dimensionless coefficient of a standardised (= centred and scaled) predictor variable in the PLS regression model |
| Bft | Bft | Beaufort wind force scale, based on the empirical relationship v /m s^-1^ = 0.836 × Bft^1.5^ (according to Beer, T. 1997. Environmental Oceanography. CRC Press. ISBN 0-8493-8425-7) |
| *DCAT* | km | the minimum distance of a shore section (represented by the *UDL10* coordinates) from the route of the catamaran (average value from both directions of travel) |
| *DWLA* | m | distance between the crest of the wrack line and the emersed structures (riparian vegetation) |
| *DWLB* | m | distance between the crest of the wrack line and the water level |
| *EF* | m | effective fetch of a shore section at the 4 m bathymetric line |
| *ES* | ° | shoreline exposure |
| *F* | m | fetch |
| *Hsig* , *Hmax* | m | significant, maximum wave height |
| *HWL* | m | average thickness of wrack near to the crest of the wrack line |
| *LW* | m NHN | long-term mean annual low water level at Lake Constance-Obersee (meteorological normal period 1990/91 to 2020/21; German ordnance datum NHN92): 394.58 m NHN |
| *LWL* | m | shore parallel length of the wrack line |
| *M* (X) |  | arithmetic mean of the variable X |
| *MCx* | % | share of the material class x in wrack composition |
| *Md*(X) |  | median of the variable X |
| *MW* | m NHN | long-term annual mean water level at Lake Constance-Obersee (meteorological normal period 1990/91 to 2020/21; German ordnance datum NHN92): 395.24 m NHN |
| *NWL* | - | number of wrack lines |
| *p* |  | level of significance |
| PLS |  | Partial Least Squares Regression |
| *SD(*X*)* |  | standard deviation of the variable X |
| *t*, \|*t*\| |  | test statistic of the paired (two-sided) t-test |
| *TEF* | m | total effective fetch of a shore section at the 4 m bathymetric line |
| *Tsig* | s | significant wave period |
| *TWE, TWE’, TWE’’* | m | total wind exposure of a shore section, based on wind data from weather station Konstanz, the nearest weather station (‘), or weighted by winds and the reciprocal of the squared distance to the weather station (“) |
| *UDL10, UDL50* | m NHN  above/below MW | vertical level of the landward limit of the submerged macrophyte vegetation when falling below a critical coverage of 10% respectively 50% |
| *VWL, Vtotal* | m^3^ | volume per running metre (i.e. specific volume) of the lower wrack line; total specific volume of all wrack lines |
| *WEU, WSUB* | m | width of the lower eulittoral zone (*MW* – *LW*) or sublittoral zone (*LW* – basin edge, 390.50 m NHN) |
| *WWEH* | % | exposure to wind waves exceeding significant wave height of *H* (cm) in % of the total time span in view (H = 5, 10, 15, 20, 25, 30 cm) |
| *WWL* | m | average width of the wrack line |
| *X%, G%, S%, UT%* | % | percentage composition of the surface sediments at the position of UDL50, based on visual estimations (X – cobbles, G – gravel, S – sand, UT – silt+clay; Wentworth classes) |
| *XG%, SUT%* |  | pooled grain size classes *X%* + *G%*, and *S%* + *UT%* |
| *YR* |  | study year (2019, 2020) |
| *Z* | m | relative level of the crest above the current lake level |
| *Zveg* | m NHN  m above or below MW | level of the lakeside border of the amphibious/terrestrial perennial vegetation (spares shore vegetation, reeds, riparian woods) |
| *ZWL_top_ ; ZWL_base_* | m NHN  m above or below MW | level of the crest of the wrack line; level of the base of a wrack line under its crest (German ordnance datum NHN92) |
